# Menstrual cycle length variation by demographic characteristics from the Apple Women’s Health Study

**DOI:** 10.1038/s41746-023-00848-1

**Published:** 2023-05-29

**Authors:** Huichu Li, Elizabeth A. Gibson, Anne Marie Z. Jukic, Donna D. Baird, Allen J. Wilcox, Christine L. Curry, Tyler Fischer-Colbrie, Jukka-Pekka Onnela, Michelle A. Williams, Russ Hauser, Brent A. Coull, Shruthi Mahalingaiah

**Affiliations:** 1 Department of Environmental Health, Harvard T.H. Chan School of Public Health, Boston, 02115 MA USA; 2 Department of Biostatistics, Harvard T.H. Chan School of Public Health, Boston, 02115 MA USA; 3 Epidemiology Branch, Division of Intramural Research, National Institute of Environmental Health Sciences, National Institutes of Health, Research Triangle Park, Durham, 27709 NC USA; 4 Health, Apple Inc., Cupertino, 95014 CA USA; 5 Department of Epidemiology, Harvard T.H. Chan School of Public Health, Boston, 02115 MA USA

**Keywords:** Reproductive signs and symptoms, Epidemiology

## Abstract

Menstrual characteristics are important signs of women’s health. Here we examine the variation of menstrual cycle length by age, ethnicity, and body weight using 165,668 cycles from 12,608 participants in the US using mobile menstrual tracking apps. After adjusting for all covariates, mean menstrual cycle length is shorter with older age across all age groups until age 50 and then became longer for those age 50 and older. Menstrual cycles are on average 1.6 (95%CI: 1.2, 2.0) days longer for Asian and 0.7 (95%CI: 0.4, 1.0) days longer for Hispanic participants compared to white non-Hispanic participants. Participants with BMI ≥ 40 kg/m^2^ have 1.5 (95%CI: 1.2, 1.8) days longer cycles compared to those with BMI between 18.5 and 25 kg/m^2^. Cycle variability is the lowest among participants aged 35–39 but are considerably higher by 46% (95%CI: 43%, 48%) and 45% (95%CI: 41%, 49%) among those aged under 20 and between 45–49. Cycle variability increase by 200% (95%CI: 191%, 210%) among those aged above 50 compared to those in the 35–39 age group. Compared to white participants, those who are Asian and Hispanic have larger cycle variability. Participants with obesity also have higher cycle variability. Here we confirm previous observations of changes in menstrual cycle pattern with age across reproductive life span and report new evidence on the differences of menstrual variation by ethnicity and obesity status. Future studies should explore the underlying determinants of the variation in menstrual characteristics.

## Introduction

Menstrual cycle characteristics, including cycle length and regularity (i.e., the variability of cycle length within an individual), have been recognized to be an important vital sign^1^. Accumulating evidence has also documented associations of long and/or irregular menstrual cycles with higher risk of infertility, cardiometabolic disease, and death^2–6^.

It has been shown that menstrual cycle length varies considerably within an individual throughout the reproductive life span^7–9^. Several studies using menstrual diary data from small numbers of individuals reported decreasing length and variability of menstrual cycle with increasing age from late adolescence/early adulthood until late reproductive age (i.e., age 40–45)^9–14^. Reports on menstrual characteristics were limited for women above age 45, and current evidence suggested menstrual cycles were increasingly longer and more varied in this age group^8,15,16^. Obesity has been linked with longer and less regular menstrual cycles, however, the results were not consistent^10,14,16,17^. The recent emergence of menstrual cycle tracking applications (apps) in smart phones allows large epidemiologic studies to confirm previous findings in small population samples and generate new evidence on factors of menstrual health^18–20^. Two studies using the app data reported similar changes of menstrual patterns by age as observed previously but results for body mass index (BMI) were still inconsistent^21,22^. In addition, adjustment for confounding, such as age, ethnicity, diet, and physical activity, was limited in these studies.

Earlier evidence on menstrual characteristics was mainly from studies among white women and has been used to establish the normal range of menstrual cycle length for clinical practice^23,24^. Separate observations among individuals in Japan, China, and India reported approximately 1–2 days longer cycle lengths compared to those observed in the white population, indicating a possibility that the suggested cycle pattern parameters may not be applicable in individuals with different ethnic backgrounds^25–27^. Other studies also found ethnic differences in hormones related to menstruation^28–31^. However, studies directly examining ethnic differences in menstrual patterns were limited and included small groups of individuals^11,14,16,32^. Therefore, evidence from a larger population is warranted.

In this study, we used menstrual cycle tracking data and survey information to confirm the associations between age and BMI with menstrual cycle length and cycle variability, and to examine possible differences of menstrual characteristics by ethnicity in a nationwide digital cohort of women within the United States (US).

## Results

### Participant characteristics

A total of 794,282 menstrual cycles from 52,117 participants enrolled in the Apple Women’s Health Study (AWHS) by December 31, 2021, were initially identified. After applying the exclusion criteria, a total of 165,668 menstrual cycles from 12,608 participants were included in the final analysis with a median of 11 cycles per participant (interquartile range, IQR = 5, 20) (Supplementary Figure 1). A total of 64,326 cycles were tracked after enrollment, and among them 85% (*N* = 54,804) were confirmed to be accurate. Approximately 88% (*N* = 11,040) of participants had at least three menstrual cycles. Mean age of eligible participants at baseline was 33 years old (SD = 8) and over 70% of the participants were white. Nearly 35% (*N* = 4379) of the participants were obese (Table 1). Distributions of other related reproductive and lifestyle factors (e.g., parity, smoking, alcohol use, physical activity, and socioeconomic status) were summarized in Supplementary Table 1. The distribution of menstrual cycle length peaked at 28 days and had a long right tail (Supplementary Figure 2, Supplementary Table 2). The mean (SD) of this distribution was 28.7 days (6.1). The median (IQR) was 28 days (26, 30 days) and the 5–95th percentile was 22–38 days. A total of 8153 (5%) long cycles and 14,976 (9%) short cycles were identified. A total of 5683 participants reported their history of COVID-19 infection, among them 4,119 reported never had known COVID-19 infection.

**Table 1:** Distribution (% and *N*) of key characteristics in AWHS.

	By participant (*N* = 12,608)	By cycle (*N* = 165,668)
*Age (years)*		
Under 20	5.2 (651)	5.9 (9735)
20–24	14.7 (1853)	13.6 (22,493)
25–29	17.2 (2169)	15.5 (25,752)
30–34	19.2 (2424)	17.5 (29,038)
35–39	18.3 (2306)	19.2 (31,793)
40–44	14.2 (1787)	16.1 (26,685)
45–49	8.4 (1065)	9.5 (15,782)
Above 50	2.8 (353)	2.6 (4390)
*Ethnicity*		
White	71.4 (8996)	71 (117,591)
Black	5.0 (633)	5.5 (9044)
Asian	4.3 (541)	4.7 (7856)
Hispanic	7.4 (928)	7.0 (11,652)
Other	2.2 (280)	2.2 (3658)
More than one ethnicity	9.8 (1230)	9.6 (15,867)
*BMI*		
Underweight	2.6 (330)	2.7 (4457)
Healthy	36.8 (4636)	37.9 (62,747)
Overweight	25.9 (3263)	25.3 (41,995)
Class 1 obese	16.8 (2112)	16.9 (28,031)
Class 2 obese	9.7 (1223)	9.2 (15,295)
Class 3 obese	8.3 (1044)	7.9 (13,143)

Values are presented as the percentage of the total participants or menstrual cycles included in this analysis.

Age was calculated as age at enrollment for the *By participant* column and as age at each cycle for the *By cycle* column.

The other ethnicity category includes American Indian or Alaska Native, Middle Eastern or North African, Native Hawaiian or Pacific Islander, or other unspecified ethnicity.

Underweight was defined as BMI < 18.5 kg/m^2^. Healthy BMI was defined as 18.5 ≤ BMI < 25 kg/m^2^. Overweight was defined as 25 ≤ BMI < 30 kg/m^2^. Class 1 obese was defined as 30 ≤ BMI < 35 kg/m^2^. Class 2 obese was defined as 35 ≤ BMI < 40 kg/m^2^. Class 3 obese was defined as BMI ≥ 40 kg/m^2^.

*AWHS* Apple Women’s Health Study, *BMI* body mass index.

### Age, ethnicity, BMI, and menstrual cycle length

**Fig. 1:**
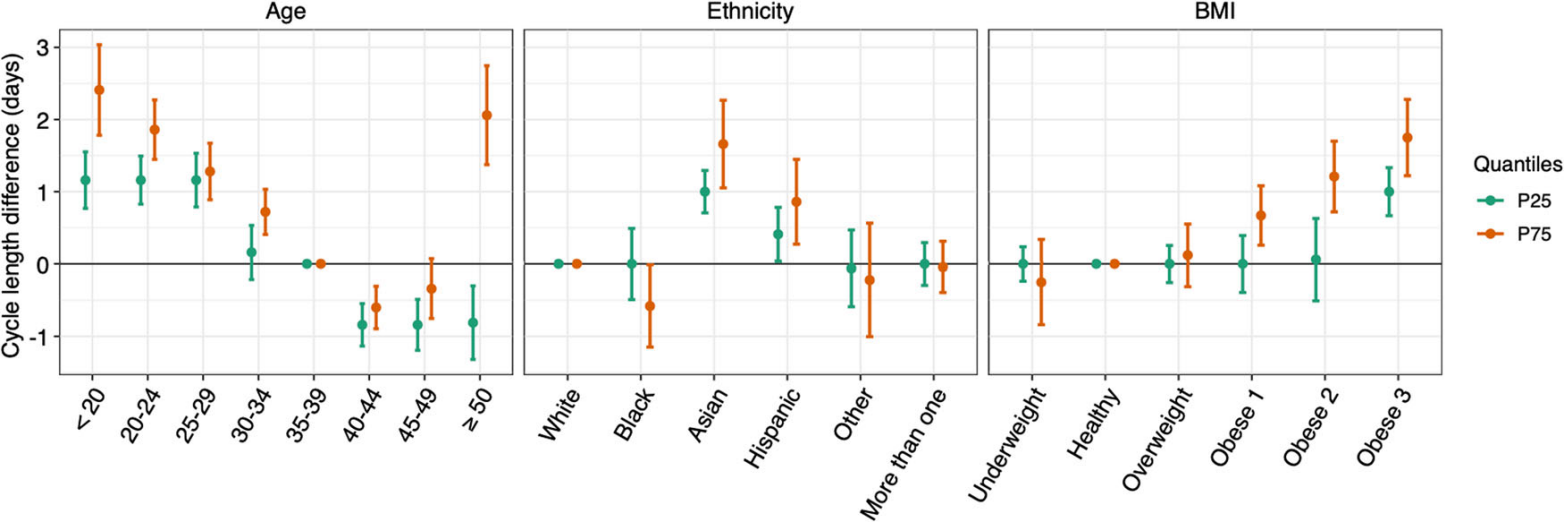
Differences and 95%CIs of menstrual cycle length on the 25th and 75th percentiles by age, ethnicity, and BMI. CIs, confidence intervals; BMI, body mass index, P25: 25th percentile; P75: 75th percentile. Exclusively adjusting for age, ethnicity, and BMI, and additionally for smoking, alcohol drinking, parity, physical activity, education, perceived stress scores, and MacArthur scale of subjective social status. Missing values in age, ethnicity, and BMI were excluded. Missing values in other covariates were treated with missing indicator. The other ethnicity group includes American Indian or Alaska Native, Middle Eastern or North African, Native Hawaiian or other Pacific Islander, or other unspecified ethnicity. Underweight: BMI < 18.5 kg/m^2^ healthy: 18.5 ≤ BMI < 25 kg/m^2^, overweight: 25 ≤ BMI < 30 kg/m^2^, class 1 obese: 30 ≤ BMI < 35 kg/m^2^, class 2 obese: 35 ≤ BMI < 40 kg/m^2^, class 3 obese: BMI ≥ 40 kg/m^2^. Error bars indicate 95%CIs.

**Fig. 2:**
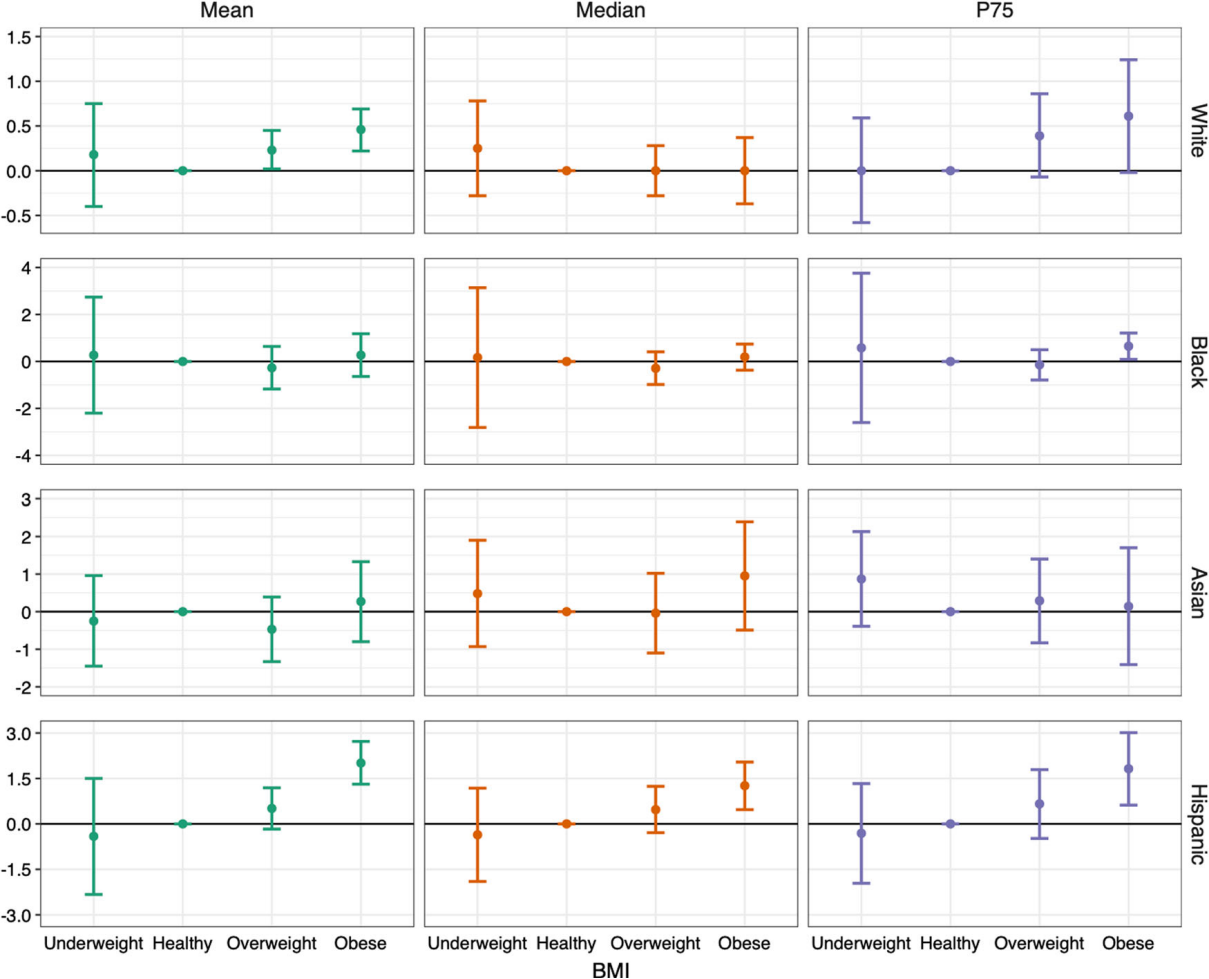
Differences and 95%CIs of the mean and median menstrual cycle length with BMI by ethnicity groups. CIs, confidence intervals; BMI, body mass index Adjusting for age, smoking, alcohol drinking, parity, physical activity, education, perceived stress scores, and MacArthur scale of subjective social status. Missing values in age, ethnicity, and BMI were excluded. Missing values in other covariates were treated with missing indicator. Analysis was restricted to participants who were under age 50 years and had BMI < 40 kg/m^2^, and to white, Black, Asian, and Hispanic participants to avoid having strata with few observations. Underweight: BMI < 18.5 kg/m^2^; healthy: 18.5 ≤ BMI < 25 kg/m^2^, overweight: 25 ≤ BMI < 30 kg/m^2^, obese: 30 ≤ BMI < 40 kg/m^2^. Error bars indicate 95%CIs.

After adjusting for all covariates, we found mean menstrual cycle length to differ by age, ethnicity, and BMI groups (Table 2). Results for age were presented using age 35–39 as the reference because this group had the lowest cycle variability. Compared to the referent group, the mean cycle length was 1.6 (95%CI: 1.3, 1.9), 1.4 (95%CI: 1.2, 1.7), 1.1 (95%CI: 0.9, 1.3) and 0.6 (95%CI: 0.4, 0.7) days longer for women aged under 20, 20–24, 25–29, and 30–34, respectively. Menstrual cycle length continued to decrease by 0.5 (95%CI: −0.3, 0.7) and 0.3 (95%CI: −0.1, 0.6) days in the 40–44 and 45–49 age groups, respectively, and increased by 2.0 (95%CI: 1.6, 2.4) days among participants above age 50. Compared to white participants, the cycles of Asian participants were 1.6 (95%CI: 1.2, 2.0) days longer and the cycles for Hispanic participants were 0.7 (95%CI: 0.4, 1.0) days longer. No notable differences were found among the remaining ethnicity groups, with cycle lengths were 0.2 (95%CI: −0.1, 0.6) days shorter in Black participants, and were 0.2 (95%CI: −0.4, 0.7) and 0.1 (95%CI: −0.2, 0.4) days longer for those in the other ethnicity group and who reported more than one ethnicity. Compared to those with healthy BMI, the cycles of those with overweight were 0.3 (95%CI: 0.1, 0.5) days longer, with Class 1 obesity were 0.5 (95%CI: 0.3, 0.8) days longer, with Class 2 obesity were 0.8 (95%CI: 0.5, 1.0) days longer, and Class 3 obesity were 1.5 (95%CI: 1.2, 1.9) days longer. The linear quantile mixed model suggested similar differences of cycle length by age, ethnicity, and BMI except that some patterns were more evident for cycle length on the 75th percentile than the median and on the 25th percentile (Table 2, Fig. 1), reflecting the skewed distribution of cycle length. No notable effect modification was found between age and ethnicity and between age and BMI (p for Wald test of interaction terms = 0.31 and 0.25). Obesity was associated with longer mean cycle length in white and Hispanic participants, while the associations were more evident for the Hispanic group (p for Wald test for interaction terms = 0.0033). An increase in cycle length with obesity for Black participants was not apparent in the mean or median estimates but was evident when the 75th percentile was examined. The differences of cycle length by BMI were moderate and statistically null in Asian participants (Fig. 2).

**Table 2:** Differences and 95% confidence intervals (95%CIs) of mean and median menstrual cycle length with age, ethnicity, and BMI in 165,668 cycles from 12,608 participants in the AWHS.

	Difference and 95% CIs of mean menstrual cycle length (days)	Difference and 95% CIs of median menstrual cycle length (days)
*Age (years)*		
Under 20	1.63 (1.31, 1.94)	1.81 (1.18, 2.44)
20–24	1.43 (1.19, 1.67)	1.38 (0.87, 1.88)
25–29	1.12 (0.91, 1.33)	1.15 (0.83, 1.47)
30–34	0.56 (0.40, 0.73)	0.62 (0.41, 0.83)
35–39	Reference	Reference
40–44	−0.49 (−0.66, −0.32)	−0.43 (−0.62, −0.23)
45–49	−0.33 (−0.56, −0.10)	−0.49 (−0.77, −0.22)
Above 50	2.02 (1.64, 2.39)	0.13 (−0.72, 0.99)
*Ethnicity*		
White	Reference	Reference
Black	−0.24 (−0.61, 0.14)	−0.04 (−0.60, 0.53)
Asian	1.57 (1.16, 1.98)	1.36 (0.85, 1.86)
Hispanic	0.73 (0.41, 1.05)	0.79 (0.29, 1.30)
Other	0.18 (−0.38, 0.73)	−0.43 (−0.88, 0.03)
More than one ethnicity	0.13 (−0.15, 0.40)	0.07 (−0.25, 0.38)
*BMI*		
Underweight	0.04 (−0.44, 0.51)	−0.01 (−0.64, 0.63)
Healthy	Reference	Reference
Overweight	0.26 (0.07, 0.45)	0.26 (−0.06, 0.57)
Class 1 obese	0.54 (0.32, 0.77)	0.21 (−0.16, 0.58)
Class 2 obese	0.76 (0.48, 1.03)	0.60 (0.18, 1.03)
Class 3 obese	1.54 (1.24, 1.85)	1.23 (0.85, 1.60)

Estimates were exclusively adjusted for age, ethnicity, and BMI, and additionally for smoking, alcohol use, parity, physical activity, education, perceived stress scores, and MacArthur scale of subjective social status. Missing values in age, ethnicity, and BMI were excluded. Missing values in other covariates were treated with missing indicator.

The other ethnicity category includes American Indian or Alaska Native, Middle Eastern or North African, Native Hawaiian or Pacific Islander, or other unspecified ethnicity.

Underweight was defined as BMI < 18.5 kg/m^2^. Healthy BMI was defined as 18.5 ≤ BMI < 25 kg/m^2^. Overweight was defined as 25 ≤ BMI < 30 kg/m^2^. Class 1 obese was defined as 30 ≤ BMI < 35 kg/m^2^. Class 2 obese was defined as 35 ≤ BMI < 40 kg/m^2^. Class 3 obese was defined as BMI ≥ 40 kg/m^2^.

*AWHS* Apple Women’s Health Study, *BMI* body mass index.

The odds of long or short cycles also differed by age, ethnicity, and BMI (Table 3). Younger participants were more likely than those aged 35–39 years to experience long cycles (under age 20 vs age 35–39: OR = 1.85, 95%CI: 1.48, 2.33; age 20–24 vs age 35–39: OR = 1.87, 95%CI: 1.56, 2.25) and less likely to have short cycles (under age 20 vs age 35–39: OR = 0.90, 95%CI: 0.74, 1.10; age 25–29 vs age 35–39: OR = 0.91, 95%CI: 0.78, 1.06), while those in 45–49 year age group were more likely to experience both long and short cycles (OR = 1.72, 95%CI: 1.41, 2.09 for long cycles and OR = 2.44, 95%CI: 2.17, 2.75 for short cycles compared to age 35–39). This trend was more evident in participants above age 50 years (OR = 6.47, 95%CI: 5.25, 7.98 for long cycles and OR = 3.25, 95%CI: 2.74, 3.86 for short cycles compared to age 35–39). Asian and Hispanic participants were more likely to have long cycles (OR = 1.43, 95%CI: 1.17, 1.75 for Asian and OR = 1.26, 95%CI: 1.07, 1.48 for Hispanic) and less likely to have short cycles (OR = 0.67, 95%CI: 0.54, 0.84 for Asian and OR = 0.87, 95%CI: 0.74, 1.02 for Hispanic) compared to white participants. Similarly, obese participants were more likely to have a long cycle (OR = 1.31, 95%CI: 1.14, 1.50 for Class 1 obesity; OR = 1.35, 95%CI: 1.14, 1.59 for Class 2 obesity and OR = 1.81, 95%CI: 1.54, 2.13 for Class 3 obesity compared to healthy BMI) but not a short cycle (Table 3). All sensitivity analyses showed similar results to the main analyses (Supplementary Tables 3–7).

**Table 3:** Odds ratios (ORs) and 95% confidence intervals (95%CIs) of experiencing a short (<24 days) or long (>38 days) menstrual cycle by age, ethnicity, and BMI in 165,668 cycles from 12,608 participants in the AWHS.

	Percentages of short cycles	OR of having a short cycle (95%CI)	Percentages of long cycles	OR of having a long cycle (95%CI)
*Age (years)*				
Under 20	8.4	0.90 (0.74, 1.10)	9.0	1.85 (1.48, 2.33)
20–24	7.7	0.91 (0.78, 1.06)	7.4	1.87 (1.56, 2.25)
25–29	6.5	0.77 (0.66, 0.88)	6.6	1.70 (1.43, 2.02)
30–34	7.0	0.83 (0.73, 0.94)	4.8	1.28 (1.08, 1.53)
35–39	8.4	Reference	3.2	Reference
40–44	12.2	1.49 (1.33, 1.68)	2.8	0.99 (0.81, 1.20)
45–49	16.7	2.44 (2.17, 2.75)	5.0	1.72 (1.41, 2.09)
Above 50	21.9	3.25 (2.74, 3.86)	18.2	6.47 (5.25, 7.98)
*Ethnicity*				
White	10.0	Reference	5.1	Reference
Black	9.3	0.97 (0.82, 1.14)	4.8	1.09 (0.89, 1.34)
Asian	7.1	0.67 (0.54, 0.84)	7.6	1.43 (1.17, 1.75)
Hispanic	6.8	0.87 (0.74, 1.02)	7.0	1.26 (1.07, 1.48)
Other	9.2	0.89 (0.69, 1.14)	4.7	1.05 (0.77, 1.44)
More than one ethnicity	8.8	1.01 (0.89, 1.16)	5.6	1.00 (0.86, 1.17)
*BMI*				
Underweight	9.4	0.98 (0.79, 1.22)	6.8	1.23 (0.94, 1.62)
Healthy	9.8	Reference	4.8	Reference
Overweight	9.4	0.92 (0.84, 1.01)	5.1	1.10 (0.98, 1.25)
Class 1 obese	9.8	0.93 (0.83, 1.03)	5.8	1.31 (1.14, 1.50)
Class 2 obese	8.5	0.78 (0.68, 0.90)	5.8	1.35 (1.14, 1.59)
Class 3 obese	8.8	0.80 (0.69, 0.92)	7.8	1.81 (1.54, 2.13)

Estimates were exclusively adjusted for age, ethnicity, and BMI, and additionally for smoking, alcohol use, parity, physical activity, education, perceived stress scores, and MacArthur scale of subjective social status. Missing values in age, ethnicity, and BMI were excluded. Missing values in other covariates were treated with missing indicator.

Long cycles were excluded from the calculation of percentage of short cycles and estimating the OR and 95%CI of having a short cycle. Likewise, short cycles were excluded from the calculation of percentage of long cycles and estimating the OR and 95%CI of having a long cycle.

The other ethnicity category includes American Indian or Alaska Native, Middle Eastern or North African, Native Hawaiian or Pacific Islander, or other unspecified ethnicity.

Underweight was defined as BMI < 18.5 kg/m^2^. Healthy BMI was defined as 18.5 ≤ BMI < 25 kg/m^2^. Overweight was defined as 25 ≤ BMI < 30 kg/m^2^. Class 1 obese was defined as 30 ≤ BMI < 35 kg/m^2^. Class 2 obese was defined as 35 ≤ BMI < 40 kg/m^2^. Class 3 obese was defined as BMI ≥ 40 kg/m^2^.

*AWHS* Apple Women’s Health Study, *BMI* body mass index.

### Age, ethnicity, BMI, and cycle variability

The average within-individual variability varied between 4–6 days across most of the age, ethnicity, and BMI groups. Changes in cycle variability with age were the most evident compared to ethnicity and BMI (Table 4). The 35–39 year age group had the lowest variability and relative increases in variability were observed in both younger and older age groups. For example, compared to the 35–39 age group, participants in the under 20, 20–24, 25–29, and 30–34 age groups had 45% (95%CI: 43, 48), 37% (34, 40), 25% (95%CI: 22, 28), and 13% (95%CI: 10, 16) higher cycle variability, respectively, and those who aged between 40–44 and 45–49 had 6% (95%CI: 1, 10) and 44% (95%CI: 40, 49) higher variability. In addition, participants over age 50 years had an estimated 200% (95%CI: 191, 209) higher variability compared to those age 35–39 years. Asian and Hispanic participants had 9% (95%CI: 3, 15) and 10% (95%CI: 3, 17) higher cycle variability compared to white participants, while no notable differences in cycle variability were found for Black participants (−3%, 95%CI: −5, 0), who reported other ethnicity (−4%, 95%CI: −9, 0), and who reported more than one ethnicity (−5%, 95%CI: −15, 4) compared to white participants. Obese participants had larger within-individual variability than those with healthy BMI, with increases of 12% (95%CI: 10, 15), 10% (95%CI: 6, 13), and 27% (95%CI: 23, 30) for those with Class 1, 2, and 3 obesity. Notably, the ORs for irregularity were the highest when comparing the age 45–49 (OR = 4.75, 95%CI: 4.46, 5.06) and age above 50 groups (OR = 27.22, 95%CI: 24.93, 29.74) compared to the age 35–39 group. Asian and Hispanic participants had 44% and 30% higher odds of experiencing irregularity (OR = 1.44, 95%CI: 1.34, 1.54 for Asian and OR = 1.30, 95%CI: 1.23, 1.38 for Hispanic) compared to white participants. Higher BMI was associated with irregularity, especially for those with Class 3 obesity (OR = 1.85, 95%CI: 1.75, 1.96 compared with the healthy BMI groups) (Table 5). Results for irregularity were similar when applying different definitions (Supplementary Table 8).

**Table 4:** Estimates and percentage change (95%CIs) of within-individual standard deviations (SDs) by age, ethnicity, and BMI in 163,275 cycles by 11,040 participants who contributed at least three complete menstrual cycles in the AWHS.

	Estimates (days)	Adjusted percentage difference (95%CIs)
*Age (years)*		
Under 20	5.33 (5.16, 5.51)	45.51 (42.82, 48.20)
20–24	5.07 (4.96, 5.18)	37.33 (34.12, 40.54)
25–29	4.70 (4.62, 4.79)	25.17 (22.24, 28.10)
30–34	4.28 (4.21, 4.34)	12.67 (9.71, 15.63)
35–39	3.79 (3.77, 3.82)	Reference
40–44	3.99 (3.94, 4.04)	5.77 (1.26, 10.28)
45–49	5.42 (5.27, 5.57)	44.54 (40.52, 48.57)
Above 50	11.19 (8.94, 13.45)	200.31 (191.10, 209.52)
*Ethnicity*		
White	4.81 (4.79, 4.83)	Reference
Black	4.67 (4.60, 4.74)	−2.55 (−5.05, −0.04)
Asian	5.04 (4.95, 5.13)	8.91 (3.09, 14.74)
Hispanic	5.09 (5.01, 5.17)	9.96 (3.18, 16.74)
Other	4.63 (4.57, 4.68)	−4.51 (−9.35, 0.34)
More than one ethnicity	4.58 (4.50, 4.66)	−5.39 (−15.31, 4.54)
*BMI*		
Underweight	4.89 (4.74, 5.03)	8.60 (2.45, 14.75)
Healthy	4.57 (4.55, 4.60)	Reference
Overweight	4.82 (4.77, 4.87)	4.30 (1.87, 6.73)
Class 1 obese	5.03 (4.96, 5.10)	12.21 (9.78, 14.63)
Class 2 obese	4.77 (4.69, 4.85)	9.82 (6.21, 13.43)
Class 3 obese	5.43 (5.32, 5.54)	26.90 (23.41, 30.38)

Within-individual cycle length standard deviation estimated from the univariate log-linear model for residual variance in a linear mixed effect model with all covariates in the fixed effect terms and participant-specific random intercepts.

Percentage difference in within-individual cycle length standard deviation estimated from the log-linear model for residual variance exclusively adjusted for age, ethnicity, and BMI. This log-linear model was embedded in a linear mixed effect model with all covariates in the fixed effect terms and participant-specific random intercepts. Missing values in age, ethnicity, and BMI were excluded. Missing values for other covariates (included in the fixed effect terms) were treated with missing indicator.

The other ethnicity category includes American Indian or Alaska Native, Middle Eastern or North African, Native Hawaiian or Pacific Islander, or other unspecified ethnicity.

Underweight was defined as BMI < 18.5 kg/m^2^. Healthy BMI was defined as 18.5 ≤ BMI < 25 kg/m^2^. Overweight was defined as 25 ≤ BMI < 30 kg/m^2^. Class 1 obese was defined as 30 ≤ BMI < 35 kg/m^2^. Class 2 obese was defined as 35 ≤ BMI < 40 kg/m^2^. Class 3 obese was defined as BMI ≥ 40 kg/m^2^.

*AWHS* Apple Women’s Health Study, *BMI* body mass index.

**Table 5:** Odds ratios (ORs) of experiencing irregularity with 95% confidence intervals (95%CIs) by age, ethnicity, and BMI in 163,275 cycles by 11,040 participants who contributed at least three complete menstrual cycles in the AWHS.

	Percentages of irregularity	OR of experiencing irregularity (95%CI)
*Age (years)*		
Under 20	20.4	2.55 (2.36, 2.76)
20–24	20.9	2.70 (2.53, 2.89)
25–29	15.9	2.03 (1.90, 2.16)
30–34	11.6	1.31 (1.23, 1.40)
35–39	10.6	Reference
40–44	12.8	1.42 (1.33, 1.52)
45–49	28.2	4.75 (4.46, 5.06)
Above 50	60.2	27.22 (24.93, 29.74)
*Ethnicity*		
White	16.5	Reference
Black	16.3	0.76 (0.71, 0.82)
Asian	18.7	1.44 (1.34, 1.54)
Hispanic	20.2	1.30 (1.23, 1.38)
Other	15.6	0.60 (0.53, 0.67)
More than one ethnicity	15.0	0.85 (0.80, 0.90)
*BMI (kg/m*^*2*^*)*		
Underweight	18.4	1.28 (1.17, 1.40)
Healthy	14.7	Reference
Overweight	15.3	1.01 (0.97, 1.06)
Class 1 obese	18.4	1.28 (1.22, 1.34)
Class 2 obese	17.6	1.15 (1.08, 1.22)
Class 3 obese	24.1	1.85 (1.75, 1.96)

Irregularity was defined as participants whose mean difference in lengths of adjacent menstrual cycles ≥7 days. the ORs and 95%CIs were exclusively adjusted for age, ethnicity, and BMI, and additionally for smoking, alcohol drinking, parity, physical activity, education, perceived stress scores, and MacArthur scale of subjective social status.

The other ethnicity category includes American Indian or Alaska Native, Middle Eastern or North African, Native Hawaiian or Pacific Islander, or other unspecified ethnicity.

Underweight was defined as BMI < 18.5 kg/m^2^. Healthy BMI was defined as 18.5 ≤ BMI < 25 kg/m^2^. Overweight was defined as 25 ≤ BMI < 30 kg/m^2^. Class 1 obese was defined as 30 ≤ BMI < 35 kg/m^2^. Class 2 obese was defined as 35 ≤ BMI < 40 kg/m^2^. Class 3 obese was defined as BMI ≥ 40 kg/m^2^.

*AWHS* Apple Women’s Health Study, *BMI* body mass index.

## Discussion

In this digital cohort study, we comprehensively examined differences in menstrual cycle length and variability by age, ethnicity, and BMI. Our results for age suggested that compared to participants in early reproductive years (i.e., under age 20 or between age 20–24), menstrual cycles were shorter for those in the older age groups up until age 50 years. Cycle length variability was the smallest among those aged 35–39 and became considerably larger among those at age 45–49 years and 50 years and above. Asian and Hispanic participants and those with higher BMI had longer cycles and higher cycle variability.

The recommended normal range of menstrual cycle length (5–95th percentiles) from FIGO was generated from reproductive age females who were menstruating and did not use hormonal medications. Compared to the FIGO normal cycle length range (24–38 days), cycle length distribution in AWHS showed a comparable 95th percentile but a shorter 5th percentile^23^. Our findings on differences in cycle length and variability by age were consistent with previous reports using data from diaries^7,8,12,33^ and mobile apps^21,22,34^. In addition, with larger sample size, our findings expanded previous knowledge on age-related changes of menstrual cycles generated from diary-based studies to a larger population. Compared to studies using data from mobile apps, we were able to capture a study population with diverse fertility needs by covering females who may nor may not actively attempt conception and control for other known factors that may explain the age-related changes in menstrual cycles such as BMI, smoking, physical activity, socioeconomic status, and stress. Our findings aligned with the menstrual cycle characteristics across reproductive life span^35^. The observed shorter cycle length in older groups under age 50 may be explained by the decreasing ovarian reserve over time, as previous studies have showed lower ovarian reserve was associated with shorter cycle length in reproductive aged women^36,37^. We found changes in the mean and median cycle length differed for those aged above 50 years. Participants who were over age 50 years had much longer and more variable cycles, as would be expected during the late menopausal transition that is characterized by highly variable cycles and frequent anovulation^35^, although studies of menstrual cycle characteristics in late menopausal stage are still limited.

Previous studies have reported ethnic differences in reproductive aging patterns and reproductive hormone levels, suggesting a possibility that menstrual cycle length and variability could differ by race and ethnicity. However, evidence is still limited and inconclusive. It has been reported that age-matched Hispanic and Asian women had higher anti-Müllerian hormone, a marker of ovarian reserve, compared to white females, but evidence was mixed for African-American individuals^31^. Other studies reported higher estrogen levels in African-American, Hispanic, and Asian females compared to those who were white, but the timing of hormone measurement within the menstrual cycle was different across these studies. A few population-based studies have compared menstrual patterns by ethnicity. One study in adolescents reported African-American girls had moderately shorter cycle length compared to European-American girls^11^. Two studies among peri-menopausal women in the US found Asian participants (Japanese and Chinese) had longer cycles compared to the white participants. In addition, Hispanic individuals were more likely to experience a cycle longer than 33 days, while no notable differences were observed between African-American individuals and those who were white^16,32^. A study of reproductive aged women in the semiconductor industry also showed longer cycle length in Asian participants compared to those who were white^14^. A recent app-based study reported longer and more irregular cycles for US Hispanic users compared to Black users^34^. In this study, we observed Asian and Hispanic participants had slightly longer cycle lengths, moderately larger within-individual cycle variability, and were more likely to experience cycle irregularity. No notable differences in cycle length and variability were found between Black and white participants. In addition, the ethnic differences of cycle length persisted after we controlled for factors that can interfere with the hypothalamus-pituitary-ovary axis, thyroids, adrenal gland, and, consequentially, menstrual cycles, such as BMI, physical activity, stress, and socioeconomic status. This indicates other unmeasured factors such as disparities in life-course exposures to cultural, social, and environmental factors may impact menstrual cycle patterns^12,17^. However, since the magnitude of the observed cycle pattern differences in our study were limited, more studies are needed to evaluate the actual impact of menstrual pattern difference on reproductive health.

Our results also suggest overweight and obese participants have longer menstrual cycles, greater cycle variability, and are more likely to experience irregularity than the healthy weight participants. Previous studies have linked higher body weight with long menstrual cycles and higher cycle variability^10,12,13^. A study among a very large number of app users reported participants with BMI between 35–50 kg/m^2^ had higher cycle variation by 0.4 days and longer mean cycle length by 0.5 days than those with a healthy BMI^21^. However, only 8% of the participants had BMI above 30 kg/m^2^. Another large app-based study found small differences in cycle length and variability across BMI groups overall, but the underweight group had higher cycle variability and a higher proportion of participants with BMI ≥ 35 kg/m^2^ had a median cycle length above 36 days^22^. Obesity has been linked with endocrine disruptions such as hyperinsulinemia and excess leptin secretion, which may affect the hormonal regulation of menstrual function^38^. Reproductive hormone profiles may also differ across BMI groups. One study suggested that compared to non-obese women, those who were obese had lower estradiol and inhibin B at premenopausal stage, while no differences were found for FSH across BMI groups^39^. Other pathways include obesity-related chronic low-grade inflammation and oxidative stress, which can adversely affect the ovary. Fat tissue is a peripheral producer of estrogen (estrone), which can affect the regulatory activity of the hypothalamic-pituitary-ovarian axis and therefore, possibly inhibits ovarian gonadotropin and estrogen production^40,41^. Obesity and long and/or irregular menstrual cycles are individually and jointly associated with cardiometabolic risk^3,4^, which has been found disproportionally higher among Hispanic and Black individuals in the US^42^. However, the associations of obesity with longer cycles were stronger in Hispanic than in Black participants in our study, though both had relatively small sample size. In addition, we excluded participants with Class 3 obesity group in the effect modification analysis to avoid subgroups with small sample sizes, which limited our ability to fully quantify the heterogenous associations of BMI and cycle length by ethnicity. Future studies could explore the possible interactions among ethnicity, obesity, and menstrual function to better understand their relationships with cardiometabolic health.

Though we had sufficient statistical power to detect minor differences in menstrual cycle length, our sample size was relatively small compared to other studies using menstrual tracking app data^18,19,21,22,34^. A notable limitation of this study is the reliance on self-reported information to measure menstrual cycles and all other covariates, with the user’s reporting/tracking behavior and health conditions possibly affecting accuracy of the study data. In the future, it would be informative to validate the self-reported information with data from other sources such as comparing self-reported medical history with clinical health records and using physical activity data collected from Apple Watch instead of self-reported physical activity from the survey. However, these data were not incorporated for this analysis due to limited data coverage corresponding to this analysis. There is no widely accepted gold standard in menstrual cycle measurement in epidemiological studies. While inaccurate reporting is still possible, cycle data collected using a prospective diary is considered more accurate than the self-reported typical cycle length and variability in surveys^43–45^. Previous studies have suggested that when height and weight are self-reported, use of the continuous measure of BMI calculated from these values could introduce less bias to the model estimates compared to the categorical BMI^46,47^. Our sensitivity analysis using continuous BMI measures showed estimates similar to those obtained from our primary analyses (Supplementary Table 9). In addition, although BMI is a common and convenient metric to identify obesity due to excess body fat, it could lead to misclassification, and the degree and direction will likely differ by ethnicity and other factors such as age and education^48,49^. Several app-based studies have included fertility awareness measures such as ovulation testing and basal body temperature measures, which allows them to consider more detailed aspects of menstruation, specifically among females avoiding or attempting conception^18,21^. However, such data were limited in this sample of AWHS participants, with approximately 5–6% of the participants reported attempting conception each month^50^. When comparing cycle characteristics across age groups, we arbitrarily used the 35–39 year age group as the reference because this group had the lowest cycle variability. Therefore, the estimates should be interpreted with considerations on the reproductive life stages to which these age groups correspond. Also, part of our study data were collected during the COVID-19 pandemic. However, our sensitivity analysis among participants who never had known COVID-19 infection showed comparable estimates to those in the main model. In addition, a separate analysis in AWHS suggests changes in menstrual cycle length associated with COVID-19 vaccination are moderate and very short-term^51^. Therefore, we believed both COVID-19 infection and vaccination had minimal impact on our results. Other factors of menstrual cycles such as sleep quality and duration were collected from Apple Watch. However, we did not consider using monitoring data in this study because the coverage was limited. Although information on race and ethnicity was collected, approximately 60% of the participants who reported Hispanic ethnicity did not report any race, which limited our ability to further compare cycle length and variability across combinations of ethnicity (Supplemental Table 10). Generalizability is also limited because our study participants are all iPhone users and those who can communicate in English, which could lead to underrepresentation of individuals with low socioeconomic status and Hispanic population.

This study quantified and examined menstrual cycle length and variability by age, ethnicity, and BMI using data collected from mobile apps in a large, diverse population in the US. Our work confirmed previous findings on changes of menstrual pattern across reproductive life span and provided new evidence for women’s health clinical practitioners to understand the extent to which menstrual patterns may vary across key characteristics. The average menstrual cycle length and variability across ethnicity and BMI groups were within the normal range and changes of cycle characteristics by age groups aligned with the natural ovarian aging process. Our findings on demographic variations have significance for epidemiologic and clinical research. More specifically, our results provided evidence on factors of menstrual cycles to inform consideration of potential confounders and/or effect modifiers for future research. As for clinical care, although the observed ethnic differences in menstrual cycle length and variability were not large enough to be clinically impactful, knowing the magnitude of such difference is important for healthcare practitioners to better provide care and consultation on menstrual health.

In addition, our analysis showed participants who were Asian or Hispanic and who had higher BMI had higher odds of menstrual irregularity, which may indicate an underlying susceptibility of gynecological disorder. Future studies should explore the underlying determinants of the variation in menstrual characteristics.

## Methods

### Study design and population

The Apple Women’s Health Study is an ongoing, prospective digital cohort study. Users of the Apple Research app on their iPhone were eligible if they have ever menstruated at least once in life, live in the US, were at least 18 years old (at least 19 in Alabama and Nebraska, and 21 in Puerto Rico), and are able to communicate in English. Eligibility also required sole usage of their iCloud account or iPhone. Enrollment began on November 2019 and is ongoing. Written informed consent of participation is provided at enrollment. This study has been approved by the Institutional Review Board at Advarra (CIRB #PRO00037562) and has been registered in Clinicaltrials.gov (NCT04196595).

Detailed information on the study design and data collection has been published^52^. Briefly, participants are asked to complete surveys on demographic characteristics (e.g., race and ethnicity, height and weight, and socioeconomic status) and reproductive history. They are also asked to self-report gynecological conditions (e.g., polycystic ovarian syndrome (PCOS), uterine fibroids, and hysterectomy) and health behaviors (e.g., smoking, alcohol use, and physical activity) which are surveyed every 12 months during follow-up. Factors related to menstrual cycles, including hormone use, pregnancy, lactation, and menopause, are collected at enrollment and updated monthly in surveys. Information on cycle tracking accuracy was also collected in monthly surveys after enrollment.

For this analysis, we included eligible AWHS participants who did not report menopause, who enrolled and contributed at least one completed menstrual cycle by December 31, 2021, and who had no history of PCOS, uterine fibroids, or hysterectomy. We excluded participants with uterine fibroids because they may be more likely to experience intermenstrual bleeding, which may affect the accuracy of menstrual cycle identification^24^.

### Menstrual cycle identification

Participants can track their menstrual flow using the Cycle Tracking feature with the Apple Health app or other third-party apps that the participant allows to write to the Health app. We collected menstrual flow entries prospectively after enrollment and any entries up to 24 months prior to enrollment. Spotting, defined as any bleeding that happens outside of the regular period, is not included in this analysis. A menstrual cycle was defined as one or more consecutive days with tracked menstrual flow followed by at least 2 days of no tracked flow. The first day of the having menstrual flow was identified as the first day of the cycle, as defined previously^8^. Cycles shorter than 10 days or longer than 90 days are excluded from the analysis as they were unlikely for a natural menstrual cycle^21^. For the remaining cycles, we excluded cycles that were atypically long and likely artifacts due to gaps in record-keeping using participant-specific thresholds modified from a previous study^19^. More specifically, these atypically long cycles were identified with an individual-specific threshold considering the typical menstrual cycle length and menstrual cycle variability of that individual. We used the median menstrual cycle length and median of the cycle length difference (calculated as the absolute value of the difference of the length between two adjacent cycles) to represent the typical menstrual cycle length and cycle variation of that person. We chose the median because the mean and standard deviation of cycle length in the raw data were more likely to be influenced by extreme values from artifacts. However, it is possible that a participant can naturally experience an atypically long cycle. Therefore, we added a 15-day window to the threshold to account for such possibility. We chose 15 days because our data suggested this as the optimal value to achieve a balance between identifying cycle artifacts and preserving natural cycle variation. The final individual-specific threshold was calculated as the sum of the median cycle length and median cycle length difference plus 15 days, and a cycle longer than this threshold value was identified as an artifact. Detecting cycle artifacts among peri-menopausal participants may be difficult because these individuals usually have large cycle variability and frequent anovulation^7,8^. Therefore, we only applied this identification in participants under age 50. For women above age 50, we included all their cycles into the analysis.

Among the 742,747 cycles within 10–90 days from 49,238 AWHS participants under age 50, a total of 29,174 cycles from 18,043 participants were identified as artifacts, which corresponds to an average of 1.62 cycles per individual. This average is comparable to the average of 1.59 cycles per user with artifacts in the previous study^19^. The distribution of median cycle length was largely unchanged before and after the exclusion, suggesting our approach only identified and excluded outliers for each individual (Supplementary Table 11). As shown in Supplementary Figure 3, there was a density peak for cycles that were approximately 21–32 days longer than median cycle length in the raw data, suggesting a possibility of cycle artifacts. After excluding these cycles, this atypical density peak disappeared. In addition, the long right tail in the histogram after exclusion suggested the natural variability of menstrual cycle length was preserved. Supplementary Figure 4 compares the distribution of menstrual cycle length among cycles that were identified as artifacts and those that were not. Most cycles identified as artifacts were within 50–60 days long, which is approximately twice the length of a typical menstrual cycle. These atypically long cycles may be artificially created when a participant missed logging a bleeding period in this interval.

For menstrual cycles tracked after enrollment, we only included those that have been confirmed with no hormone use, pregnancy, or lactation in the monthly surveys. For cycles tracked prior to enrollment, we only included those from participants who confirmed none of these events in the previous 2 years.

### Measurement of age, ethnicity, and BMI

Age was calculated as the difference between the year of the first day of the cycle and the participant’s birth year and was categorized as under 20, 20–24, 25–29, 30–34, 35–39, 40–44, 45–49, and above 50 years. Race and ethnicity were self-reported by participants, using the following pre-specified categories (defined by researchers) in the survey with instructions to check all that apply: white, non-Hispanic (referred to as ‘white’); Black or African American or African (referred to as ‘Black’); Asian; Hispanic, Latino, Spanish and/or other Hispanic (referred to as ‘Hispanic’); American Indian or Alaska Native; Middle Eastern or North African; Native Hawaiian or Pacific Islander; and an option indicating that none of these categories can fully describe the participant. For this analysis, we combined participants who were American Indian or Alaska Native, Middle Eastern or North African, Native Hawaiian or Pacific Islander, or who indicated none of these categories can fully describe the participant into one group because of small numbers. Participants who chose more than one category were combined in a separate group. Body mass index (BMI) was calculated using the self-reported height and weight. This was categorized as underweight (BMI < 18.5 kg/m^2^), healthy (18.5 ≤ BMI < 25 kg/m^2^), overweight (25 ≤ BMI < 30 kg/m^2^), and obese (BMI ≥ 30 kg/m^2^). The obese group was further divided into Class 1 (30 ≤ BMI < 35 kg/m^2^), 2 (35 ≤ BMI < 40 kg/m^2^), and 3 (BMI ≥ 40 kg/m^2^)^53^.

### Measurement of other covariates

We considered possible confounders or predictors of menstrual cycle length, including cigarette smoking (never smoked, previously smoked, and currently smoke), alcohol use, physical activity, stress, socioeconomic status, and parity (nulliparous and parous). All covariates were self-reported through surveys. Alcohol use was measured as frequency of up to once a month, 2–4 times a month, 2–3 times a week, and more than 4 times a week. Physical activity was categorized as none, light (e.g., walking or light housework), moderate (e.g., brisk walking or yard work), vigorous (e.g., running or carrying heavy loads), and strenuous (e.g., competitive sports or endurance events like marathons). Stress was measured using the 4-item Perceived Stress Score and categorized by quartiles^54^. Socioeconomic status (SES) was measured using an objective and a subjective measure because it has been suggested that both measures could affect overall health jointly and independently^55^. Highest education level was used as an objective measure of SES. The variable included levels: high school graduate or less (in the U.S. high school is grades 9–12), 3-year college or technical schools (typically done after graduating high school, not required for entrance to a 4-year college), 4-year college degree (typically begun directly after high school), and graduate school (including master and doctoral studies; occurs after receiving an undergraduate degree). The subjective measure of SES was measured by the MacArthur scale of subjective social status. This scale is a self-rated rank on a ‘social ladder’ from 0 (lowest) to 9 (highest) based on the responder’s self-perceived education, socioeconomic status, and current life circumstances relative to others. For this analysis, we categorized this scale into low (0–3), moderate (4–6), and high (7–9).

Each participant’s tracked menstrual cycles were merged with covariate values collected from the most recent surveys prior to that cycle. Menstrual cycles tracked prior to enrollment were assigned covariate values that were reported in their first surveys, corresponding to the assumption that these covariates remained unchanged during this interval. Participants with missing information on age, ethnicity, and BMI were excluded. Missingness in the other covariates was treated with missing indicators.

### Statistical analysis

We estimated the distribution of menstrual cycle length in our study population using a Gaussian kernel density function with weights equal to the inverse of the total number of cycles contributed by that participant to avoid bias by the varying numbers of menstrual cycles per participant.

### Analysis of menstrual cycle length

We used linear mixed effect (LME) models with random participant-specific intercepts to estimate the differences and 95% confidence intervals (95%CIs) in mean menstrual cycle length by age, ethnicity, and BMI. We fitted a model exclusively adjusted for age, ethnicity, BMI, smoking, alcohol use, parity, physical activity, education, perceived stress scores, and MacArthur scale of subjective social status. We fitted a linear mixed quantile regression model for the median cycle length with age, ethnicity, and BMI, adjusted for all other covariates to examine the impact of cycle length distribution on the LME estimates^56^. To better understand how the distribution of cycle length varies with these factors, we additionally considered the 25th (P25) and 75th percentiles (P75) of cycle length in the mixed quantile regression model. Pair-wise effect modification of age, ethnicity, and BMI was considered by adding multiplicative terms in the models. Wald tests were conducted to determine the statistical significance of effect modification. We restricted the effect modification analysis to participants who were under age 50 years and had BMI < 40 kg/m^2^, and to white, Black, Asian, and Hispanic participants to avoid having strata with few cycles (Supplementary Table 11). We then categorized menstrual cycles into long (>38 days) and short (<24 days) cycles using recommendations from the International Federation of Gynecology and Obstetrics (FIGO)^23^, and examined the associations of each primary characteristic with the probability of experiencing a long or short menstrual cycle using logistic regression. The reference category for long and short cycles is menstrual cycles between 24–38 days. Participants with short cycles can contribute more cycles than those with long cycles, resulting in biased estimates from generalized estimating equations (due to informative cluster size). To address this bias, we estimated the odds ratio (OR) and 95%CI of experiencing a long or short cycle from logistic regression using the within-cluster resampling approach, adjusting for all covariates^57^. The details of within-cluster resampling has been published elsewhere^57^. Briefly, we first randomly sampled one cycle per participant with replacement from the original menstrual cycle data. In this data sample, each participant contributed only one observation and the observations are no longer correlated. Therefore, we can fit a logistic regression model for the binary outcome (i.e., experiencing a long or a short cycle) and obtain estimates and standard errors for the regression coefficients. Then we repeated the sampling and analysis steps multiple times and recorded the coefficient estimates and standard errors from each iteration. The final estimates of the regression coefficient is the mean of the coefficient estimates across all iterations. The standard error of the final coefficient estimate ${SE}\left(\hat{\beta }\right)$ is given by Eq. (1) below:

1 $$\,{SE}\left(\hat{\beta }\right)=\frac{\mathop{\sum }\nolimits_{q}^{Q}{({{SE}}_{q})}^{2}}{Q}-\,\frac{\left(Q-1\right)}{Q}{S}^{2}$$

Where ${{SE}}_{q}$ is the standard error of the coefficient from the *q*th *(q* = *1,2,…, Q)* iteration. ${S}^{2}$ is the variance of the coefficient estimates across all *Q* iterations.

It has been recommended to perform many iterations to ensure the analysis had sufficient resamples, although no recommendations on the minimum number of iterations are given. We repeated the resampling procedure for *Q* = 1000, 5000, 7500, and 10,000 iterations in preliminary analysis and the estimates and standard errors were similar (data not shown). Therefore, we presented results using 10,000 iterations. The ORs and 95%CIs for experiencing a long and short cycle estimated by within-cluster resampling were similar to those estimated using logistic regression with GEE (data not shown).

Several sensitivity analyses were considered. We repeated the analysis among participants who contributed at least three menstrual cycles because the individual-specific threshold may not effectively identify artifacts if a participant contributed very few cycles. Information on cycle tracking accuracy and most covariates was only available after enrollment. Therefore, we repeated the analysis by restricting to cycles tracked after enrollment with confirmed accuracy to examine the impact of possible measurement errors. An accurately tracked cycle was defined as a cycle started in a month when the participant responded ‘yes, they were accurate’ to the question ‘are all your period days during the previous calendar month accurately reflected in the Health app?’ in the corresponding monthly survey. We considered a complete case analysis for possible bias from missing indicators. We also repeated the analysis by including participants with uterine fibroids (*N* = 705 participants). Since part of our data were collected during the COVID-19 pandemic, we considered a sensitivity analysis restricting to those who reported never having had a known COVID-19 infection in a 2022 survey.

### Analysis of cycle length variability and irregularity

Considering women with few cycles may not effectively contribute information on cycle variability, we restricted this analysis to participants with at least three menstrual cycles. In the linear mixed model framework, within-individual variability in cycle length, which represents the degree of cycle irregularity, can be estimated by the standard deviations (SDs) of the model residuals after accounting for the systematic variability across subgroups in the fixed effect terms and between individual variability in random intercepts^8^. To quantify and examine the associations of age, ethnicity, and BMI with within-individual cycle variability, we constructed log-linear models for residual variance in the fully adjusted LME models. We first considered univariable models to estimate within-individual cycle variability (in days) by each factor. Then we fitted a multivariable model with all three variables included to obtain the adjusted estimates for the associations of age, ethnicity, and BMI with within-individual cycle variability. Coefficients from the multivariable model were computed as the percentage change in within-individual variability in cycle length compared to the referent for the factors of interest.

We further identified cycle irregularity as participants whose mean difference in lengths of adjacent menstrual cycles ≥7 days^34^ and examined the associations of age, ethnicity, and BMI with cycle irregularity using logistic regression models with iterative reweighted least squares, adjusted for all covariates. We repeated the analysis using alternative criteria of irregularity as the median difference in lengths of adjacent menstrual cycles ≥9 days^19^, the standard deviation of menstrual cycle length ≥7 days, and the difference between the shortest and longest cycle ≥7 days.

Data management, processing, and statistical analyses were conducted in R (version 3.6.0) and Python (version 3.6). All statistical tests were two-sided.

### Reporting summary

Further information on research design is available in the Nature Research Reporting Summary linked to this article.

## Supplementary information

Supplemental Materials

Reporting Summary

## Data Availability

Aggregated deidentified data that support the findings of this study may be available upon request from the corresponding author (SM). Any request for data will be evaluated and responded to in a manner consistent with policies intended to protect participant confidentiality and language in the Study protocol and informed consent form.

## Code availability

All the code that support the findings of this study may be available upon request from the corresponding author (SM).
