# Supplementary material for: Menstrual cycle length variation by demographic characteristics from the Apple Women’s Health Study
Source: NPJ Digit Med. 2023 May 29;6:100. doi: 10.1038/s41746-023-00848-1 (PMC10226714; doi:10.1038/s41746-023-00848-1)
Supplement: Supplementary file 1 — Supplemental Materials [file 41746_2023_848_MOESM1_ESM.docx]

**Supplementary Information**

**Supplementary Table 1 Distribution (% and N) of smoking, alcohol use, parity, physical activity, education, stress, and social status scale in AWHS**

|  | By participant (N=12,608) | By cycle  (N=165,668) |
| --- | --- | --- |
| Cigarette smoking |  |  |
| Never smoked | 68.2 (8,603) | 69.4 (115,013) |
| Previously smoked | 16.8 (2,115) | 17.8 (29,499) |
| Currently smoke | 10.0 (1,264) | 8.9 (14,756) |
| Missing | 5.0 (626) | 3.9 (6,400) |
| Alcohol use |  | |
| None | 15.6 (1,967) | 16.1 (26,657) |
| ≤ once a month | 32.3 (4,076) | 32.3 (53,579) |
| 2-4 times a month | 23.4 (2,956) | 23.3 (38,632) |
| 2-3 times a week | 14.9 (1,877) | 14.8 (24,569) |
| ≥ 4 times a week | 10.4 (1,308) | 10.9 (17,996) |
| Missing | 3.4 (424) | 2.6 (4,235) |
| Parity |  |  |
| Nulliparous | 55.0 (6,936) | 57.2 (94,802) |
| Parous | 37.6 (4,740) | 37.1 (61,442) |
| Missing | 7.4 (932) | 5.7 (9,424) |
| Physical activity |  |  |
| None | 2.7 (340) | 2.6 (4,324) |
| Light | 26.8 (3,375) | 26.8 (44,477) |
| Moderate | 36.0 (4,538) | 36.4 (60,341) |
| Vigorous | 28.4 (3,580) | 28.6 (47,437) |
| Strenuous | 3.0 (382) | 3.2 (5,233) |
| Missing | 3.1 (393) | 2.3 (3,856) |
| Education |  |  |
| High school graduate or less | 12.8 (1,616) | 11.9 (19,633) |
| 3-year college or technical schools | 30.0 (3,783) | 29.0 (48,045) |
| 4-year college degree | 32.9 (4,145) | 33.9 (56,117) |
| Graduate degree | 24.0 (3,032) | 25.1 (41,573) |
| Missing | 0.3 (32) | 0.2 (300) |
| 4-item Perceived Stress Score |  | |
| 1^st^ quartile (0-3) | 22.9 (2,882) | 24.8 (41,019) |
| 2^nd^ quartile (4-6) | 27.8 (3,505) | 28.0 (46,331) |
| 3rd quartile (7-9) | 26.9 (3,390) | 26.8 (44,461) |
| 4th quartile (10-16) | 19.1 (2,409) | 17.9 (29,699) |
| Missing | 3.3 (422) | 2.5 (4,158) |
| MacArthur scale of subjective social status |  | |
| Low (0-3) | 23.6 (2,977) | 22.2 (36,781) |
| Medium (4-6) | 61.3 (7,723) | 62.0 (102,690) |
| High (7-9) | 15.1 (1,898) | 15.8 (26,136) |
| Missing | 0.1 (10) | 0.0 (61) |

Abbreviations: AWHS, Apple Women’s Health Study; BMI, body mass index.

Values are presented as the percentage of the total participants or menstrual cycles included in this analysis.

Light physical activity was defined as participating in activities such as walking and light housework. Moderate physical activity was defined as participating in activities such as brisk walking or yard work. Vigorous activity was defined as participating in activities such as running or carrying heavy loads. Strenuous activity was defined as participating in activities such as competitive sports or endurance events like marathons.

**Supplementary Table 2 Numbers of menstrual cycles across different cycle length groups by age, ethnicity, and BMI in 165,668 menstrual cycles from 12,608 participants of AWHS**

|  | <24 days  (N=14,976) | 24-26 days  (N=40,663) | 27-29 days  (N=57,221) | 30-32 days  (N=28,095) | 33-35 days  (N=11,419) | - 1. Days   (N=5,141) | >38 days  (N=8,153) |
| --- | --- | --- | --- | --- | --- | --- | --- |
| Age |  |  |  |  |  |  |  |
| Under 20 | 751 | 1,433 | 2,915 | 2,057 | 1,167 | 601 | 811 |
| 20-24 | 1,623 | 3,433 | 7,436 | 5,024 | 2,340 | 1,090 | 1,547 |
| 25-29 | 1,562 | 4,595 | 9,130 | 5,492 | 2,343 | 1,044 | 1,586 |
| 30-34 | 1,949 | 6,801 | 10,508 | 5,458 | 2,113 | 906 | 1,303 |
| 35-39 | 2,581 | 9,289 | 11,974 | 4,696 | 1,656 | 667 | 930 |
| 40-44 | 3,174 | 8,876 | 9,287 | 3,240 | 1,027 | 420 | 661 |
| 45-49 | 2,517 | 5,152 | 4,935 | 1,650 | 576 | 287 | 665 |
| Above 50 | 819 | 1,084 | 1,036 | 478 | 197 | 126 | 650 |
| Ethnicity |  |  |  |  |  |  |  |
| White | 11,264 | 29,955 | 40,745 | 19,175 | 7,655 | 3,330 | 5,467 |
| Black | 806 | 2,621 | 3,142 | 1,323 | 511 | 246 | 395 |
| Asian | 517 | 1,364 | 2,558 | 1,667 | 774 | 420 | 556 |
| Hispanic | 737 | 2,335 | 4,095 | 2,248 | 984 | 487 | 766 |
| Other | 322 | 936 | 1,235 | 662 | 252 | 95 | 156 |
| More than one ethnicity | 1,330 | 3,452 | 5,446 | 3,020 | 1,243 | 563 | 813 |
| BMI |  |  |  |  |  |  |  |
| Underweight | 393 | 926 | 1,457 | 855 | 379 | 169 | 278 |
| Healthy | 5,892 | 15,332 | 22,096 | 10,736 | 4,150 | 1,834 | 2,707 |
| Overweight | 3,778 | 10,500 | 14,824 | 7,043 | 2,673 | 1,231 | 1,946 |
| Class 1 obese | 2,613 | 7,130 | 9,412 | 4,618 | 1,929 | 860 | 1,469 |
| Class 2 obese | 1,228 | 3,724 | 5,292 | 2,546 | 1,177 | 518 | 810 |
| Class 3 obese | 1,072 | 3,051 | 4,140 | 2,297 | 1,111 | 529 | 943 |

Abbreviations: AWHS, Apple Women’s Health Study; BMI, body mass index.

The other ethnicity category includes American Indian or Alaska Native, Middle Eastern or North African, Native Hawaiian or Pacific Islander, or other unspecified ethnicity.

Underweight was defined as BMI<18.5 kg/m^2^. Healthy BMI was defined as 18.5≤BMI<25 kg/m^2^. Overweight was defined as 25≤BMI<30 kg/m^2^. Class 1 obese was defined as 30≤BMI<35kg/m^2^. Class 2 obese was defined as 35≤BMI<40 kg/m^2^. Class 3 obese was defined as BMI≥40 kg/m^2^.

**Supplementary Table 3 Differences and 95% confidence intervals (95%CIs) of mean menstrual cycle length with age, ethnicity, and BMI after restricting to participants who tracked ≥ 3 cycles, to participants under age 50, to cycles with confirmed accurate tracking, to cycles with complete data, and including participants with uterine fibroids**

|  | Restricted to participants with ≥ 3 cycles | Restricted to participants under age 50 | Restricted to cycles with accurate tracking | Restricted to cycles with complete data | Including participants with uterine fibroids |
| --- | --- | --- | --- | --- | --- |
| N cycles | 163,261 | 161,278 | 54,804 | 150,197 | 175,703 |
| Age |  |  |  |  |  |
| Under 20 | 1.59 (1.29, 1.90) | 1.58 (1.27, 1.89) | 1.79 (1.25, 2.34) | 1.47 (1.14, 1.81) | 1.67 (1.36, 1.99) |
| 20-24 | 1.39 (1.15, 1.63) | 1.40 (1.16, 1.63) | 1.71 (1.36, 2.05) | 1.43 (1.18, 1.68) | 1.47 (1.23, 1.70) |
| 25-29 | 1.09 (0.89, 1.29) | 1.09 (0.89, 1.29) | 1.57 (1.27, 1.87) | 1.13 (0.92, 1.35) | 1.13 (0.93, 1.33) |
| 30-34 | 0.55 (0.38, 0.71) | 0.55 (0.39, 0.71) | 0.71 (0.45, 0.98) | 0.56 (0.38, 0.73) | 0.58 (0.42, 0.75) |
| 35-39 | Reference | Reference | Reference | Reference | Reference |
| 40-44 | -0.47 (-0.64, -0.31) | -0.51 (-0.67, -0.35) | -0.54 (-0.81, -0.27) | -0.43 (-0.61, -0.26) | -0.49 (-0.66, -0.33) |
| 45-49 | -0.31 (-0.53, -0.08) | -0.48 (-0.71, -0.25) | -0.44 (-0.77, -0.10) | -0.29 (-0.53, -0.05) | -0.30 (-0.52, -0.09) |
| Above 50 | 1.94 (1.57, 2.31) | - | 2.02 (1.49, 2.54) | 2.02 (1.63, 2.40) | 2.00 (1.65, 2.34) |
| Ethnicity |  |  |  |  |  |
| White | Reference | Reference | Reference | Reference | Reference |
| Black | -0.30 (-0.67, 0.06) | -0.26 (-0.64, 0.12) | -0.56 (-1.01, -0.11) | -0.38 (-0.78, 0.01) | -0.20 (-0.54, 0.15) |
| Asian | 1.62 (1.23, 2.01) | 1.62 (1.21, 2.04) | 1.19 (0.71, 1.67) | 1.52 (1.09, 1.95) | 1.49 (1.09, 1.89) |
| Hispanic | 0.80 (0.49, 1.11) | 0.73 (0.41, 1.05) | 0.67 (0.28, 1.06) | 0.72 (0.39, 1.05) | 0.77 (0.46, 1.07) |
| Other | -0.03 (-0.57, 0.51) | 0.30 (-0.26, 0.86) | -0.42 (-1.08, 0.24) | -0.09 (-0.69, 0.51) | 0.25 (-0.29, 0.79) |
| More than one ethnicity | 0.19 (-0.08, 0.46) | 0.16 (-0.12, 0.44) | 0.26 (-0.06, 0.58) | 0.04 (-0.25, 0.33) | 0.15 (-0.12, 0.43) |
| BMI |  |  |  |  |  |
| Underweight | 0.11 (-0.35, 0.57) | 0.02 (-0.45, 0.50) | 0.94 (0.28, 1.59) | 0.17 (-0.33, 0.67) | 0.03 (-0.44, 0.51) |
| Healthy | Reference | Reference | Reference | Reference | Reference |
| Overweight | 0.27 (0.09, 0.46) | 0.22 (0.03, 0.41) | 0.17 (-0.07, 0.41) | 0.29 (0.10, 0.49) | 0.23 (0.04, 0.41) |
| Class 1 obese | 0.51 (0.29, 0.73) | 0.57 (0.34, 0.79) | 0.55 (0.27, 0.83) | 0.54 (0.31, 0.78) | 0.57 (0.35, 0.79) |
| Class 2 obese | 0.77 (0.50, 1.04) | 0.75 (0.48, 1.03) | 0.77 (0.43, 1.12) | 0.74 (0.45, 1.03) | 0.76 (0.49, 1.03) |
| Class 3 obese | 1.50 (1.20, 1.79) | 1.49 (1.18, 1.79) | 1.51 (1.13, 1.89) | 1.59 (1.28, 1.91) | 1.57 (1.27, 1.87) |

Abbreviations: BMI, body mass index.

Estimates were exclusively adjusted for age, ethnicity, and BMI, and additionally for smoking, alcohol use, parity, physical activity, education, perceived stress scores, and MacArthur scale of subjective social status. Missing values in age, ethnicity, and BMI were excluded. Missing values in other covariates were treated with missing indicator.

The other ethnicity category includes American Indian or Alaska Native, Middle Eastern or North African, Native Hawaiian or Pacific Islander, or other unspecified ethnicity.

Underweight was defined as BMI<18.5 kg/m^2^. Healthy BMI was defined as 18.5≤BMI<25 kg/m^2^. Overweight was defined as 25≤BMI<30 kg/m^2^. Class 1 obese was defined as 30≤BMI<35 kg/m^2^. Class 2 obese was defined as 35≤BMI<40 kg/m^2^. Class 3 obese was defined as BMI≥40 kg/m^2^.

**Supplementary Table 4 Differences and 95% confidence intervals (95%CIs) of median menstrual cycle length with age, ethnicity, and BMI to participants who tracked ≥ 3 cycles, to participants under age 50, to cycles with confirmed accurate tracking, to cycles with complete data, and including participants with uterine fibroids**

|  | Restricted to participants with ≥ 3 cycles | Restricted to participants under age 50 | Restricted to cycles with accurate tracking | Restricted to cycles with complete data | Including participants with uterine fibroids |
| --- | --- | --- | --- | --- | --- |
| N cycles | 163,261 | 161,278 | 54,804 | 150,197 | 175,703 |
| Age |  |  |  |  |  |
| Under 20 | 1.98 (1.42, 2.54) | 1.97 (1.56, 2.37) | 1.87 (1.33, 2.41) | 1.89 (1.32, 2.46) | 1.93 (1.46, 2.40) |
| 20-24 | 1.54 (1.01,2.07) | 1.49 (1.05, 1.94) | 1.72 (1.41, 2.03) | 1.57 (1.01, 2.12) | 1.56 (1.12, 2.00) |
| 25-29 | 1.08 (0.73, 1.42) | 1.00 (0.73, 1.27) | 1.36 (1.01, 1.70) | 1.13 (0.80 1.45) | 1.27 (1.00, 1.53) |
| 30-34 | 0.54 (0.28, 0.80) | 0.49 (0.23, 0.76) | 0.74 (0.47, 1.01) | 0.57 (0.33, 0.80) | 0.71 (0.50, 0.91) |
| 35-39 | Reference | Reference | Reference | Reference | Reference |
| 40-44 | -0.46 (-0.66, -0.26) | -0.51 (-0.70, -0.31) | -0.56 (-0.92, 0.20) | -0.43 (-0.63, -0.23) | -0.37 (-0.54, -0.19) |
| 45-49 | -0.46 (-0.72, -0.21) | -0.51 (-0.75, -0.26) | -0.53 (-0.88, -0.18) | -0.43 (-0.70, -0.17) | -0.37 (-0.57, -0.16) |
| Above 50 | 0.55 (-0.22, 1.33) | - | 0.22 (-0.41, 0.85) | 1.01 (0.38, 1.64) | 0.77 (0.18, 1.35) |
| Ethnicity |  |  |  |  |  |
| White | Reference | Reference | Reference | Reference | Reference |
| Black | 0.00 (-0.57, 0.57) | 0.00 (-0.49, 0.49) | -0.58 (-1.00, -0.16) | -0.12 (-0.69, 0.46) | 0.00 (-0.45, 0.45) |
| Asian | 1.38 (0.77, 1.98) | 1.48 (1.01, 1.95) | 1.07 (0.59, 1.56) | 1.12 (0.54, 1.69) | 1.22 (0.83, 1.61) |
| Hispanic | 0.54 (-0.05, 1.13) | 1.00 (0.43, 1.57) | 0.55 (0.17, 0.93) | 0.66(0.08, 1.24) | 0.78 (0.22, 1.34) |
| Other | -0.46 (-0.95, 0.03) | -0.49 (-1.04, -0.05) | -0.46 (-1.01, 0.08) | -0.45 (-0.95, 0.05) | -0.30 (-0.85, 0.25) |
| More than one ethnicity | 0.00 (-0.43, 0.43) | 0.00 (-0.29, 0.29) | 0.22 (-0.08, 0.52) | 0.00 (-0.35 0.35) | 0.07 (-0.21, 0.35) |
| BMI |  |  |  |  |  |
| Underweight | 0.00 (-0.46, 0.46) | -0.01 (-0.45, 0.43) | 0.90 (0.37, 1.43) | 0.29 (-0.34, 0.93) | 0.08 (-0.39, 0.54) |
| Healthy | Reference | Reference | Reference | Reference | Reference |
| Overweight | 0.46 (0.07, 0.86) | 0.49 (0.21, 0.77 | 0.27 (-0.02, 0.56) | 0.44 (0.08, 0.79) | 0.22 (-0.05, 0.49) |
| Class 1 obese | 0.46 (0.04, 0.88) | 0.49 (0.11, 0.88) | 0.32 (0.07, 0.57) | 0.44 (0.02, 0.86) | 0.29 (-0.04, 0.62) |
| Class 2 obese | 0.53 (0.10, 0.95) | 0.99 (0.56, 1.42) | 0.58 (0.25, 0.92) | 0.57 (0.16, 0.97) | 0.66 (0.33, 0.99) |
| Class 3 obese | 1.46 (1.03, 1.89) | 1.49 (1.13, 1.86) | 1.15 (0.65, 1.65) | 1.44 (1.01, 1.87) | 1.29 (1.01, 1.58) |

Abbreviations: BMI, body mass index.

Estimates were exclusively adjusted for age, ethnicity, and BMI, and additionally for smoking, alcohol use, parity, physical activity, education, perceived stress scores, and MacArthur scale of subjective social status. Missing values in age, ethnicity, and BMI were excluded. Missing values in other covariates were treated with missing indicator.

The other ethnicity category includes American Indian or Alaska Native, Middle Eastern or North African, Native Hawaiian or Pacific Islander, or other unspecified ethnicity.

Underweight was defined as BMI<18.5 kg/m^2^. Healthy BMI was defined as 18.5≤BMI<25 kg/m^2^. Overweight was defined as 25≤BMI<30 kg/m^2^. Class 1 obese was defined as 30≤BMI<35 kg/m^2^. Class 2 obese was defined as 35≤BMI<40 kg/m^2^. Class 3 obese was defined as BMI≥40 kg/m^2^.

**Supplementary Table 5 Odds ratios (ORs) and 95% confidence intervals (95%CIs) of experiencing a short (<24 days) menstrual cycle by age, ethnicity, and BMI to participants who tracked ≥ 3 cycles, to participants under age 50, to cycles with confirmed accurate tracking, to cycles with complete data, and including participants with uterine fibroids**

|  | Restricted to participants with ≥ 3 cycles | Restricted to participants under age 50 | Restricted to cycles with accurate tracking | Restricted to cycles with complete data | Including participants with uterine fibroids |
| --- | --- | --- | --- | --- | --- |
| N short cycles/N total | 14,796/155,433 | 14,157/153,775 | 5,171/52,593 | 14,976/157,515 | 16,366/167,127 |
| Age |  |  |  |  |  |
| Under 20 | 0.96 (0.79, 1.16) | 0.90 (0.73, 1.10) | 0.79 (0.56, 1.11) | 0.93 (0.75, 1.15) | 0.87 (0.72, 1.06) |
| 20-24 | 0.97 (0.84, 1.12) | 0.91 (0.79, 1.06) | 0.78 (0.63, 0.97) | 0.86 (0.73, 1.01) | 0.90 (0.78, 1.04) |
| 25-29 | 0.75 (0.65, 0.85) | 0.77 (0.67, 0.88) | 0.72 (0.59, 0.87) | 0.74 (0.64, 0.86) | 0.77 (0.67, 0.88) |
| 30-34 | 0.83 (0.73, 0.93) | 0.83 (0.73, 0.94) | 0.84 (0.71, 1.00) | 0.82 (0.72, 0.94) | 0.83 (0.74, 0.93) |
| 35-39 | Reference | Reference | Reference | Reference | Reference |
| 40-44 | 1.52 (1.37, 1.70) | 1.49 (1.34, 1.67) | 1.48 (1.27, 1.71) | 1.50 (1.33, 1.69) | 1.51 (1.36, 1.68) |
| 45-49 | 2.45 (2.18, 2.75) | 2.46 (2.19, 2.77) | 2.47 (2.10, 2.90) | 2.40 (2.12, 2.73) | 2.40 (2.14, 2.68) |
| Above 50 | 3.33 (2.83, 3.91) | - | 3.43 (2.76, 4.27) | 3.33 (2.79, 3.97) | 3.31 (2.84, 3.85) |
| Ethnicity |  |  |  |  |  |
| White | Reference | Reference | Reference | Reference | Reference |
| Black | 0.97 (0.83, 1.13) | 0.98 (0.82, 1.16) | 1.05 (0.83, 1.33) | 0.99 (0.83, 1.17) | 0.97 (0.84, 1.12) |
| Asian | 0.69 (0.56, 0.85) | 0.67 (0.54, 0.84) | 0.82 (0.62, 1.09) | 0.67 (0.53, 0.85) | 0.68 (0.56, 0.84) |
| Hispanic | 0.79 (0.68, 0.92) | 0.87 (0.75, 1.03) | 0.79 (0.63, 1.00) | 0.87 (0.73, 1.04) | 0.87 (0.75, 1.01) |
| Other | 0.98 (0.77, 1.25) | 0.85 (0.66, 1.10) | 0.89 (0.63, 1.27) | 0.94 (0.71, 1.23) | 0.86 (0.68, 1.10) |
| More than one ethnicity | 0.95 (0.84, 1.08) | 1.00 (0.88, 1.15) | 1.03 (0.87, 1.23) | 1.06 (0.93, 1.22) | 1.01 (0.89, 1.15) |
| BMI |  |  |  |  |  |
| Underweight | 1.00 (0.82, 1.22) | 0.99 (0.80, 1.22) | 1.07 (0.77, 1.48) | 0.89 (0.71, 1.13) | 0.97 (0.78, 1.20) |
| Healthy | Reference | Reference | Reference | Reference | Reference |
| Overweight | 0.90 (0.82, 0.99) | 0.91 (0.83, 1.00) | 0.94 (0.83, 1.06) | 0.90 (0.81, 0.99) | 0.94 (0.86, 1.03) |
| Class 1 obese | 0.91 (0.82, 1.01) | 0.95 (0.85, 1.05) | 0.97 (0.84, 1.12) | 0.90 (0.81, 1.01) | 0.91 (0.82, 1.01) |
| Class 2 obese | 0.83 (0.73, 0.95) | 0.78 (0.69, 0.90) | 0.82 (0.68, 0.98) | 0.76 (0.66, 0.87) | 0.79 (0.70, 0.90) |
| Class 3 obese | 0.85 (0.73, 0.97) | 0.80 (0.69, 0.93) | 0.86 (0.71, 1.05) | 0.74 (0.64, 0.86) | 0.80 (0.70, 0.91) |

Abbreviations: BMI, body mass index.

Estimates were exclusively adjusted for age, ethnicity, and BMI, and additionally for smoking, alcohol use, parity, physical activity, education, perceived stress scores, and MacArthur scale of subjective social status. Missing values in age, ethnicity, and BMI were excluded. Missing values in other covariates were treated with missing indicator.

Long cycles were excluded when estimating the OR and 95%CI of having a short cycle.

The other ethnicity category includes American Indian or Alaska Native, Middle Eastern or North African, Native Hawaiian or Pacific Islander, or other unspecified ethnicity.

Underweight was defined as BMI<18.5 kg/m^2^. Healthy BMI was defined as 18.5≤BMI<25 kg/m^2^. Overweight was defined as 25≤BMI<30 kg/m^2^. Class 1 obese was defined as 30≤BMI<35kg/m^2^. Class 2 obese was defined as 35≤BMI<40 kg/m^2^. Class 3 obese was defined as BMI≥40 kg/m^2^.

**Supplementary Table 6 Odds ratios (ORs) and 95% confidence intervals (95%CIs) of experiencing a long (>38 days) menstrual cycle by age, ethnicity, and BMI, after restricting to women who tracked ≥ 3 cycles, to women under age 50, to cycles with confirmed accurate tracking, to cycles with complete data, and including participants with uterine fibroids**

|  | Restricted to participants with ≥ 3 cycles | Restricted to participants under age 50 | Restricted to cycles with accurate tracking | Restricted to cycles with complete data | Including participants with uterine fibroids |
| --- | --- | --- | --- | --- | --- |
| N long cycles/N total | 7,828/148,465 | 7,503/153,775 | 2,396/49,818 | 8,153/150,692 | 8,591/159,337 |
| Age |  |  |  |  |  |
| Under 20 | 2.02 (1.61, 2.54) | 1.78 (1.42, 2.24) | 1.95 (1.34, 2.84) | 1.78 (1.40, 2.26) | 1.83 (1.46, 2.29) |
| 20-24 | 2.03 (1.69, 2.44) | 1.83 (1.53, 2.19) | 1.87 (1.44, 2.42) | 1.81 (1.49, 2.20) | 1.85 (1.55, 2.21) |
| 25-29 | 1.76 (1.48, 2.10) | 1.67 (1.41, 1.99) | 1.75 (1.38, 2.22) | 1.69 (1.41, 2.02) | 1.67 (1.41, 1.98) |
| 30-34 | 1.33 (1.12, 1.58) | 1.28 (1.08, 1.52) | 1.32 (1.04, 1.67) | 1.30 (1.08, 1.55) | 1.27 (1.08, 1.51) |
| 35-39 | Reference | Reference | Reference | Reference | Reference |
| 40-44 | 0.99 (0.82, 1.21) | 0.99 (0.82, 1.20) | 0.92 (0.71, 1.19) | 1.04 (0.85, 1.27) | 0.98 (0.82, 1.18) |
| 45-49 | 2.05 (1.68, 2.49) | 1.84 (1.51, 2.23) | 1.68 (1.29, 2.19) | 1.76 (1.43, 2.16) | 1.71 (1.42, 2.05) |
| Above 50 | 6.93 (5.64, 8.53) | - | 6.78 (5.10, 9.02) | 6.59 (5.30, 8.19) | 6.44 (5.30, 7.82) |
| Ethnicity |  |  |  |  |  |
| White | Reference | Reference | Reference | Reference | Reference |
| Black | 0.97 (0.79, 1.20) | 1.07 (0.87, 1.33) | 0.87 (0.63, 1.20) | 1.00 (0.80, 1.25) | 1.11 (0.92, 1.35) |
| Asian | 1.51 (1.23, 1.85) | 1.46 (1.18, 1.80) | 1.34 (1.00, 1.81) | 1.42 (1.14, 1.77) | 1.40 (1.15, 1.72) |
| Hispanic | 1.28 (1.10, 1.51) | 1.25 (1.07, 1.46) | 1.33 (1.05, 1.69) | 1.27 (1.07, 1.50) | 1.27 (1.09, 1.49) |
| Other | 0.95 (0.69, 1.30) | 1.08 (0.78, 1.48) | 0.90 (0.56, 1.44) | 0.95 (0.67, 1.34) | 1.08 (0.80, 1.47) |
| More than one ethnicity | 0.97 (0.84, 1.14) | 1.01 (0.86, 1.18) | 1.16 (0.93, 1.45) | 1.00 (0.85, 1.18) | 1.01 (0.87, 1.17) |
| BMI |  |  |  |  |  |
| Underweight | 1.29 (0.99, 1.68) | 1.23 (0.93, 1.62) | 1.96 (1.34, 2.85) | 1.32 (0.98, 1.77) | 1.22 (0.93, 1.60) |
| Healthy | Reference | Reference | Reference | Reference | Reference |
| Overweight | 1.14 (1.01, 1.29) | 1.08 (0.95, 1.22) | 1.05 (0.88, 1.26) | 1.16 (1.02, 1.32) | 1.11 (0.98, 1.24) |
| Class 1 obese | 1.33 (1.16, 1.52) | 1.32 (1.15, 1.52) | 1.38 (1.14, 1.68) | 1.32 (1.14, 1.52) | 1.30 (1.14, 1.49) |
| Class 2 obese | 1.41 (1.20, 1.65) | 1.35 (1.14, 1.60) | 1.48 (1.18, 1.86) | 1.34 (1.13, 1.60) | 1.35 (1.16, 1.59) |
| Class 3 obese | 1.99 (1.69, 2.34) | 1.76 (1.49, 2.08) | 2.07 (1.63, 2.62) | 1.78 (1.50, 2.12) | 1.80 (1.54, 2.11) |

Abbreviations: BMI, body mass index.

Estimates were exclusively adjusted for age, ethnicity, and BMI, and additionally for smoking, alcohol use, parity, physical activity, education, perceived stress scores, and MacArthur scale of subjective social status. Missing values in age, ethnicity, and BMI were excluded. Missing values in other covariates were treated with missing indicator.

Short cycles were excluded when estimating the OR and 95%CI of having a long cycle.

The other ethnicity category includes American Indian or Alaska Native, Middle Eastern or North African, Native Hawaiian or Pacific Islander, or other unspecified ethnicity.

Underweight was defined as BMI<18.5 kg/m^2^. Healthy BMI was defined as 18.5≤BMI<25 kg/m^2^. Overweight was defined as 25≤BMI<30 kg/m^2^. Class 1 obese was defined as 30≤BMI<35kg/m^2^. Class 2 obese was defined as 35≤BMI<40 kg/m^2^. Class 3 obese was defined as BMI≥40 kg/m^2^.

**Supplementary Table 7 Differences and 95% confidence intervals (95%CIs) of mean menstrual cycle length with age, ethnicity, and BMI in 59,431 cycles from 4,119 AWHS participants who never had known COVID-19 infection**

|  | Participants, N (%) | Cycles, N (%) | Differences and 95%CIs of mean menstrual cycle length (days) | Differences and 95%CIs of median menstrual cycle length (days) |
| --- | --- | --- | --- | --- |
| Age (years) |  |  |  |  |
| Under 20 | 166 (4.0) | 2,407 (4.0) | 1.30 (0.71, 1.89) | 1.54 (0.78, 2.29) |
| 20-24 | 458 (11.1) | 6,390 (10.8) | 1.37 (0.95, 1.79) | 1.53 (0.94, 2.12) |
| 25-29 | 688 (16.7) | 8,613 (14.5) | 1.23 (0.88, 1.59) | 1.32 (0.92, 1.73) |
| 30-34 | 786 (19.1) | 10,446 (17.6) | 0.75 (0.46, 1.04) | 0.85 (0.55, 1.16) |
| 35-39 | 774 (18.8) | 11,301 (19.0) | Reference | Reference |
| 40-44 | 637 (15.5) | 10,634 (17.9) | -0.51 (-0.78, -0.24) | -0.44 (-0.65, -0.22) |
| 45-49 | 454 (11.0) | 7,564 (12.7) | -0.40 (-0.76, -0.04) | -0.40 (-0.77. -0.02) |
| Above 50 | 156 (3.8) | 2,076 (3.5) | 2.37 (1.80, 2.93) | 0.80 (-0.09, 1.70) |
| Ethnicity |  |  |  |  |
| White | 3,036 (73.7) | 43,759 (73.6) | Reference | Reference |
| Black | 207 (5.0) | 3,112 (5.2) | -0.04 (-0.69, 0.61) | -0.14 (-0.88, 0.60) |
| Asian | 192 (4.7) | 3,033 (5.1) | 1.41 (0.74, 2.08) | 1.42 (0.70, 2.13) |
| Hispanic | 247 (6.0) | 3,493 (5.9) | 0.84 (0.25, 1.43) | 1.14 (0.32, 1.97) |
| Other | 87 (2.1) | 1,274 (2.1) | 0.25 (-0.70, 1.21) | 0.14 (-0.66, 0.94) |
| More than one ethnicity | 350 (8.5) | 4,760 (8.0) | 0.11 (-0.39, 0.62) | -0.14 (-0.60, 0.31) |
| BMI |  |  |  |  |
| Underweight | 99 (2.4) | 1,508 (2.5) | 0.46 (-0.33, 1.25) | 0.57 (-0.10, 1.24) |
| Healthy | 1,541 (37.4) | 22,691 (38.2) | Reference | Reference |
| Overweight | 1,075 (26.1) | 15,445 (26.0) | 0.23 (-0.08, 0.54) | 0.19 (-0.24, 0.62) |
| Class 1 obese | 700 (17.0) | 9,804 (16.5) | 0.62 (0.25, 0.99) | 0.45 (0.03, 0.87) |
| Class 2 obese | 389 (9.4) | 5,422 (9.1) | 0.84 (0.38, 1.31) | 1.16 (0.57, 1.75) |
| Class 3 obese | 315 (7.6) | 4,561 (7.7) | 1.64 (1.11, 2.16) | 1.28 (0.87, 1.69) |

Abbreviations: AWHS, Apple Women’s Health Study; BMI, body mass index.

Participants who never had known COVID-19 infection were identified by self-reported not having been previous tested positive for COVID-19 in 2022.

All models were exclusively adjusted for age, ethnicity, and BMI, and additionally for smoking, alcohol use, parity, physical activity, education, perceived stress scores, and MacArthur scale of subjective social status. Missing values in age, ethnicity, and BMI were excluded. Missing values in other covariates were treated with missing indicator.

The other ethnicity category includes American Indian or Alaska Native, Middle Eastern or North African, Native Hawaiian or Pacific Islander, or other unspecified ethnicity.

Underweight was defined as BMI<18.5 kg/m^2^. Healthy BMI was defined as 18.5≤BMI<25 kg/m^2^. Overweight was defined as 25≤BMI<30 kg/m^2^. Class 1 obese was defined as 30≤BMI<35kg/m^2^. Class 2 obese was defined as 35≤BMI<40 kg/m^2^. Class 3 obese was defined as BMI≥40 kg/m^2^.

**Supplementary Table 8 Odds ratios (ORs) and 95% confidence intervals (95%CIs) of experiencing menstrual irregularity by age, ethnicity, and BMI using different criteria among 11,040 participants with ≥ 3 menstrual cycles in AWHS**

|  | Standard deviation of cycle length ≥7 days | Median cycle length difference ≥9 days | Longest and shortest cycle differ by ≥7 days |
| --- | --- | --- | --- |
| Age |  |  |  |
| Under 20 | 2.83 (2.60, 3.08) | 2.06 (1.82, 2.33) | 3.36 (3.11, 3.63) |
| 20-24 | 3.00 (2.80, 3.22) | 2.47 (2.24, 2.74) | 1.74 (1.65, 1.83) |
| 25-29 | 1.97 (1.84, 2.11) | 2.05 (1.86, 2.25) | 1.46 (1.39, 1.52) |
| 30-34 | 1.56 (1.46, 1.67) | 1.52 (1.38, 1.68) | 1.12 (1.07, 1.16) |
| 35-39 | Reference | Reference | Reference |
| 40-44 | 1.43 (1.34, 1.54) | 1.41 (1.27, 1.56) | 1.25 (1.20, 1.30) |
| 45-49 | 5.11 (4.78, 5.47) | 4.54 (4.13, 5.00) | 2.52 (2.37, 2.67) |
| Above 50 | 37.80 (34.50, 41.44) | 22.09 (19.81, 24.63) | 5.75 (4.96, 6.71) |
| Ethnicity |  |  |  |
| White | Reference | Reference | Reference |
| Black | 0.91 (0.84, 0.98) | 0.73 (0.65, 0.82) | 0.78 (0.74, 0.83) |
| Asian | 1.27 (1.18, 1.38) | 1.08 (0.96, 1.20) | 1.18 (1.11, 1.26) |
| Hispanic | 1.41 (1.33, 1.50) | 1.39 (1.28, 1.51) | 1.06 (1.00, 1.12) |
| Other | 0.67 (0.58, 0.75) | 0.78 (0.66, 0.92) | 0.93 (0.85, 1.01) |
| More than one ethnicity | 0.86 (0.81, 0.91) | 0.84 (0.77, 0.91) | 0.99 (0.94, 1.03) |
| BMI |  |  |  |
| Underweight | 1.40 (1.27, 1.53) | 1.19 (1.03, 1.37) | 1.22 (1.12, 1.34) |
| Healthy | Reference | Reference | Reference |
| Overweight | 1.06 (1.01, 1.10) | 1.26 (1.19, 1.34) | 1.03 (1.00, 1.07) |
| Class 1 obese | 1.24 (1.18, 1.30) | 1.36 (1.27, 1.46) | 1.07 (1.03, 1.11) |
| Class 2 obese | 1.29 (1.21, 1.37) | 1.41 (1.30, 1.54) | 1.16 (1.10, 1.22) |
| Class 3 obese | 1.71 (1.61, 1.83) | 1.91 (1.76, 2.08) | 1.41 (1.34, 1.49) |

Abbreviations: AWHS, Apple Women’s Health Study; BMI, body mass index.

Estimates were exclusively adjusted for age, ethnicity, and BMI, and additionally for smoking, alcohol use, parity, physical activity, education, perceived stress scores, and MacArthur scale of subjective social status. Missing values in age, ethnicity, and BMI were excluded. Missing values in other covariates were treated with missing indicator.

Short cycles were excluded when estimating the OR and 95%CI of having a long cycle.

The other ethnicity category includes American Indian or Alaska Native, Middle Eastern or North African, Native Hawaiian or Pacific Islander, or other unspecified ethnicity.

Underweight was defined as BMI<18.5 kg/m2. Healthy BMI was defined as 18.5≤BMI<25 kg/m2. Overweight was defined as 25≤BMI<30 kg/m2. Class 1 obese was defined as 30≤BMI<35kg/m2. Class 2 obese was defined as 35≤BMI<40 kg/m2. Class 3 obese was defined as BMI≥40 kg/m^2^.

**Supplementary Table 9 Differences and 95% confidence intervals of mean menstrual cycle length by BMI groups using categorical, continuous, and piecewise BMI**

|  | Median BMI  (kg/m^2^) | Difference in mean menstrual cycle length | | |
| --- | --- | --- | --- | --- |
|  |  | Main model  (Categorical BMI) | Continuous BMI | Piecewise BMI |
| Underweight | 17.8 | 0.04 (-0.44, 0.51) | 0.27 (0.18, 0.36) | 0.04 (-14.5, 14.5) |
| Healthy | 22.3 | Reference | Reference | Reference |
| Overweight | 27.3 | 0.26 (0.07, 0.45) | 0.27 (0.18, 0.36) | 0.27 (0.18, 0.36) |
| Obese 1 | 32.1 | 0.54 (0.32, 0.77) | 0.56 (0.38, 0.74) | 0.56 (0.38, 0.74) |
| Obese 2 | 37.2 | 0.76 (0.48, 1.03) | 0.86 (0.58, 1.14) | 0.86 (0.58, 1.14) |
| Obese 3 | 44.1 | 1.54 (1.24, 1.85) | 1.28 (0.86, 1.70) | 1.28 (0.86, 1.70) |

Abbreviations: BMI, body mass index.

All models were adjusted for age, ethnicity, additionally for smoking, alcohol use, parity, physical activity, education, perceived stress scores, and MacArthur scale of subjective social status. The difference and 95% confidence intervals by BMI categories in the continuous and piecewise BMI models were calculated using the differences of the median BMI in each category and the one in the healthy BMI category.

Underweight was defined as BMI<18.5 kg/m2. Healthy BMI was defined as 18.5≤BMI<25 kg/m2. Overweight was defined as 25≤BMI<30 kg/m2. Class 1 obese was defined as 30≤BMI<35kg/m2. Class 2 obese was defined as 35≤BMI<40 kg/m2. Class 3 obese was defined as BMI≥40 kg/m^2^.

Piecewise BMI model was fitted by adding a multiplicative interaction term of being underweight (binary) and the continuous BMI in the model with all other covariates remained the same. We considered this model because the main model with categorical BMI suggested potential non-linear relationship of BMI and mean menstrual cycle length.

**Supplementary Table 10 Cycle length difference and 95% confidence intervals (95%CIs) by ethnicity in Apple Women’s Health Study**

|  | Number of participants | Number of menstrual cycles | Mean cycle length difference and 95%CIs from age adjusted model | Mean cycle length difference and 95%CIs from fully adjusted model |
| --- | --- | --- | --- | --- |
| White, Non-Hispanic | 8996 | 117591 | Reference | Reference |
| White, Hispanic | 427 | 5432 | -0.01 (-0.46, 0.45) | -0.09 (-0.54, 0.37) |
| Asian, Hispanic | 12 | 165 | 1.10 (-1.53, 3.72) | 0.85 (-1.75, 3.45) |
| Asian, Non-Hispanic | 541 | 7856 | 1.38 (0.98, 1.79) | 1.57 (1.16, 2.38) |
| Black, Hispanic | 53 | 766 | 0.70 (-0.54, 1.95) | 0.41 (-0.83, 1.65) |
| Black, Non-Hispanic | 633 | 9044 | 0.04 (-0.33, 0.42) | -0.24 (-0.61, 0.14) |
| Missing, Hispanic | 928 | 11652 | 0.92 (0.60, 1.24) | 0.73 (0.41, 1.05) |
| Multiple, Hispanic | 110 | 1492 | -0.05 (-0.92, 0.83) | -0.12 (-0.99, 0.75) |
| Multiple, Non-Hispanic | 594 | 7641 | 0.35 (-0.04, 0.74) | 0.29 (-0.09, 0.68) |
| Other, Hispanic | 34 | 371 | 0.27 (-1.31, 1.85) | -0.04 (-1.60, 1.52) |
| Other, Non-Hispanic | 101 | 1477 | -0.32 (-1.24, 0.59) | -0.38 (-1.29, 0.52) |
| None of these categories can fully describe me | 179 | 2181 | 0.61 (-0.09, 1.31) | 0.50 (-0.19, 1.20) |

Estimates were exclusively adjusted for age, ethnicity, and BMI, and additionally for smoking, alcohol use, parity, physical activity, education, perceived stress scores, and MacArthur scale of subjective social status. Missing values in age, ethnicity, and BMI were excluded. Missing values in other covariates were treated with missing indicator.

The other ethnicity category includes American Indian or Alaska Native, Middle Eastern or North African, Native Hawaiian or Pacific Islander, or other unspecified ethnicity.

**Supplementary Table 11 Distributions of median cycle length and median cycle length difference before and after excluding artifacts, and threshold values among 49,238 participants under age 50 of AWHS**

|  | | | P10 | | P25 | | P50 | | P75 | | P90 | |
| --- | --- | --- | --- | --- | --- | --- | --- | --- | --- | --- | --- | --- |
| Median cycle length |  | |  | |  | |  | |  | |  |  |
| Before | | | 25 | | 27 | | 28.5 | | 31.5 | | 37 | |
| After | | | 25 | | 26.5 | | 28 | | 30 | | 33 | |
| Median cycle length difference | | |  | |  | |  | |  | |  | |
| Before | | | 1 | | 2 | | 3 | | 7 | | 16 | |
| After | | | 1 | | 2 | | 3 | | 6 | | 14 | |
| Threshold value for artifacts | | | 43 | | 44 | | 47 | | 53 | | 67 | |

Abbreviation: AWHS, Apple Women’s Health Study

**Supplementary Table 12 Number of menstrual cycles by age and BMI across ethnicity groups in 165,668 menstrual cycles from 12,608 participants of AWHS**

|  | White  (N=117,951) | Black  (N=9,044) | Asian  (N=7,856) | Hispanic  (N=11,652) | Other  (N=3,658) | More than one  (N=15,867) |
| --- | --- | --- | --- | --- | --- | --- |
| Age |  |  |  |  |  |  |
| Under 20 | 5,865 | 627 | 504 | 1,129 | 244 | 1,366 |
| 20-24 | 14,142 | 1,307 | 1,541 | 1,916 | 635 | 2,952 |
| 25-29 | 17,159 | 1,429 | 1,708 | 2,062 | 680 | 2,714 |
| 30-34 | 20,900 | 1,475 | 1,179 | 2,255 | 798 | 2,431 |
| 35-39 | 23,706 | 1,705 | 1,128 | 1,989 | 474 | 2,791 |
| 40-44 | 20,093 | 1,544 | 1,004 | 1,544 | 482 | 2,018 |
| 45-49 | 12,144 | 781 | 604 | 709 | 241 | 1,303 |
| Above 50 | 3,582 | 176 | 188 | 48 | 104 | 292 |
| BMI |  |  |  |  |  |  |
| Underweight | 2,907 | 150 | 586 | 358 | 49 | 407 |
| Healthy | 45,357 | 2,090 | 4,640 | 3,592 | 1,503 | 5,565 |
| Overweight | 29,730 | 2,286 | 1,764 | 3,257 | 1,027 | 3,931 |
| Class 1 obese | 19,243 | 1,819 | 644 | 2,471 | 665 | 3,189 |
| Class 2 obese | 10,917 | 1,346 | 216 | 1,144 | 274 | 1,398 |
| Class 3 obese | 9,437 | 1,353 | 6 | 830 | 140 | 1,377 |

Abbreviations: AWHS, Apple Women’s Health Study; BMI, body mass index.

The other ethnicity category includes American Indian or Alaska Native, Middle Eastern or North African, Native Hawaiian or Pacific Islander, or other unspecified ethnicity.

Underweight was defined as BMI<18.5 kg/m2. Healthy BMI was defined as 18.5≤BMI<25 kg/m^2^. Overweight was defined as 25≤BMI<30 kg/m2. Class 1 obese was defined as 30≤BMI<35kg/m^2^. Class 2 obese was defined as 35≤BMI<40 kg/m^2^. Class 3 obese was defined as BMI≥40 kg/m^2^.

**Supplementary Figure 1 Inclusion and exclusion of menstrual cycles and AWHS participants**

Age criteria: above age 18 for most states, above age 19 for Alabama and Nebraska, above 21 for Puerto Rico

Abbreviations: AWHS, Apple Women’s Health Study; BMI, body mass index


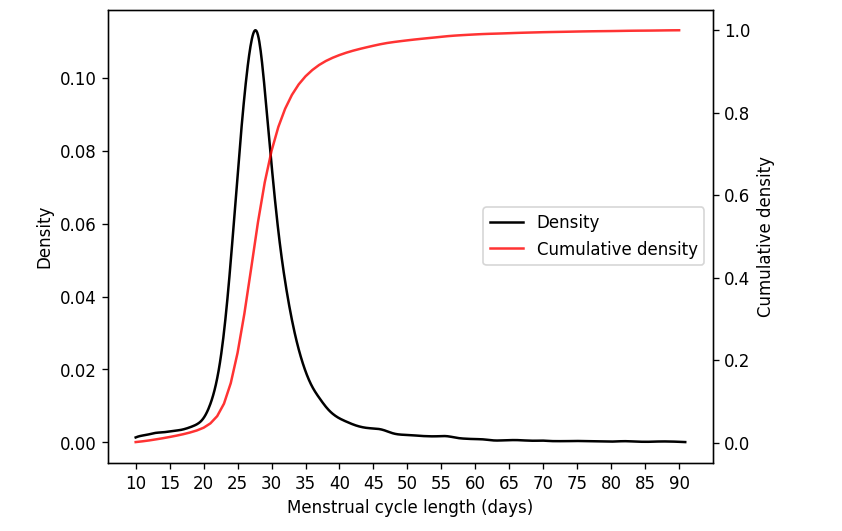


**Supplementary Figure 2 By-participant distribution (density and cumulative density) of menstrual cycle length across 165,668 cycles from 12,608 participants in the AWHS**

Density and cumulative density were estimated by weighting the cycles with the inverse of the total number of cycles contributed by each participant.

Abbreviation: AWHS, Apple Women’s Health Study.


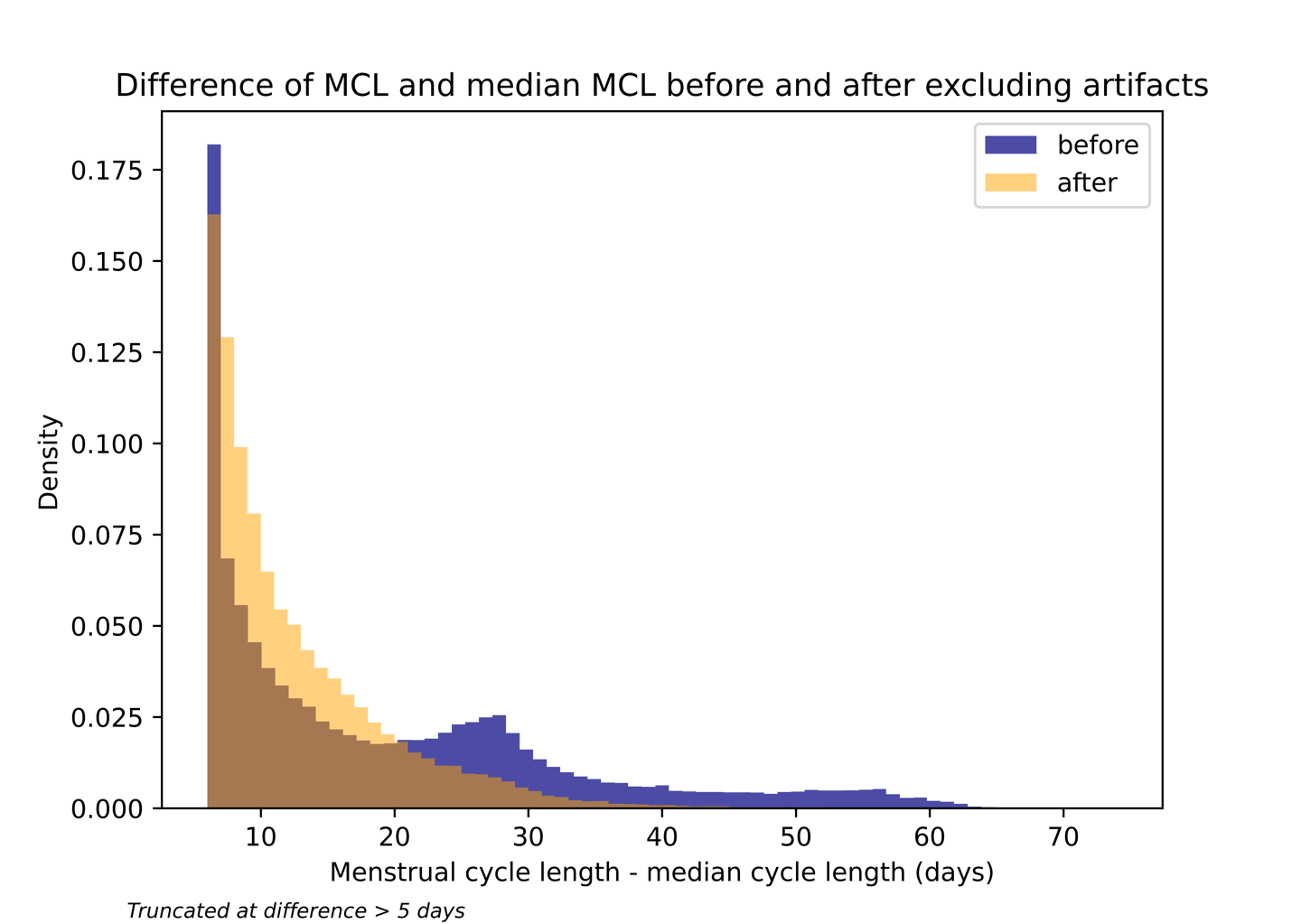


**Supplementary Figure 3 Histogram of the difference between the length of each menstrual cycle to the median cycle length of that individual before and after excluding cycle artifacts.**

Started from difference of 5 days.


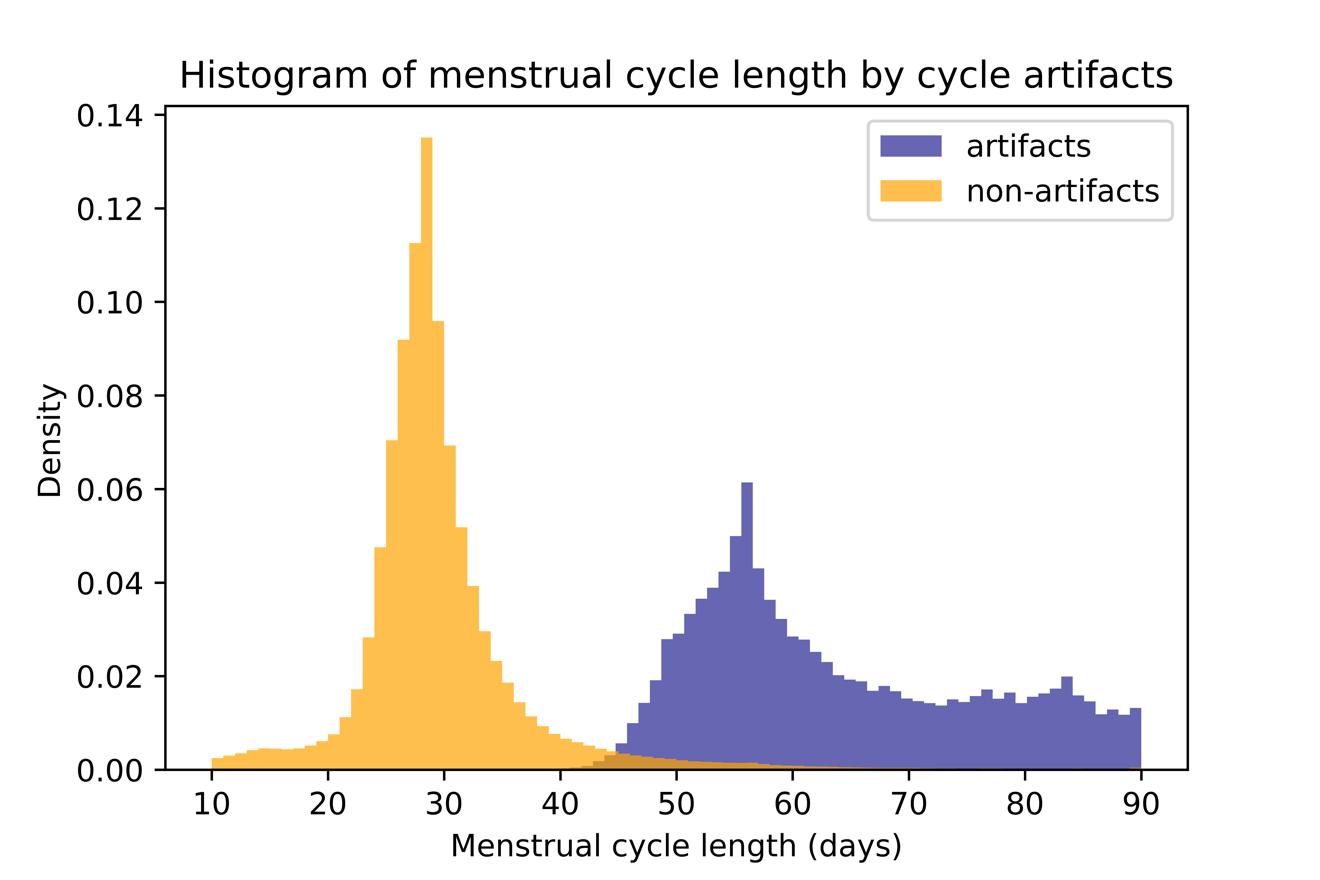


**Supplementary Figure 4 Histograms of cycle length among cycles that were identified as potential artifacts and those that were not.**
