# Supplementary material for: Menstrual cycle length variation by demographic characteristics from the Apple Women’s Health Study
Source: NPJ Digit Med. 2023 May 29;6:100. doi: 10.1038/s41746-023-00848-1 (PMC10226714; doi:10.1038/s41746-023-00848-1)
Supplement: Supplementary file 2 — Reporting Summary [file 41746_2023_848_MOESM2_ESM.pdf]

nature portfolio

Corresponding author(s): Shruthi Mahalingaiah
Last updated by author(s): May 12, 2023

Reporting Summary

Nature Portfolio wishes to improve the reproducibility of the work that we publish. This form provides structure for consistency and transparency in reporting. For further information on Nature Portfolio policies, see our Editorial Policies and the Editorial Policy Checklist.

Statistics

For all statistical analyses, confirm that the following items are present in the figure legend, table legend, main text, or Methods section.

n/a	Confirmed	
☐	☒	The exact sample size (*n*) for each experimental group/condition, given as a discrete number and unit of measurement
☐	☒	A statement on whether measurements were taken from distinct samples or whether the same sample was measured repeatedly
☐	☒	The statistical test(s) used AND whether they are one- or two-sided<br>*Only common tests should be described solely by name; describe more complex techniques in the Methods section.*
☐	☒	A description of all covariates tested
☐	☒	A description of any assumptions or corrections, such as tests of normality and adjustment for multiple comparisons
☐	☒	A full description of the statistical parameters including central tendency (e.g. means) or other basic estimates (e.g. regression coefficient) AND variation (e.g. standard deviation) or associated estimates of uncertainty (e.g. confidence intervals)
☐	☒	For null hypothesis testing, the test statistic (e.g. *F*, *t*, *r*) with confidence intervals, effect sizes, degrees of freedom and *P* value noted<br>*Give P values as exact values whenever suitable.*
☒	☐	For Bayesian analysis, information on the choice of priors and Markov chain Monte Carlo settings
☐	☒	For hierarchical and complex designs, identification of the appropriate level for tests and full reporting of outcomes
☐	☒	Estimates of effect sizes (e.g. Cohen's *d*, Pearson's *r*), indicating how they were calculated

Our web collection on statistics for biologists contains articles on many of the points above.

Software and code

Policy information about availability of computer code

Data collection	All data were collected from Apple Health App
Data analysis	Data management, processing, and statistical analyses were conducted in R (version 3.6.0) using packages 'nlme', 'lme4', and 'lqmm', and Python (version 3.6). All statistical tests were two-sided.

For manuscripts utilizing custom algorithms or software that are central to the research but not yet described in published literature, software must be made available to editors and reviewers. We strongly encourage code deposition in a community repository (e.g. GitHub). See the Nature Portfolio guidelines for submitting code & software for further information.

Data

Policy information about availability of data

All manuscripts must include a data availability statement. This statement should provide the following information, where applicable:

- Accession codes, unique identifiers, or web links for publicly available datasets
- A description of any restrictions on data availability
- For clinical datasets or third party data, please ensure that the statement adheres to our policy

Aggregated deidentified data that support the findings of this study may be available upon request from the corresponding author (SM). Any request for data will be

evaluated and responded to in a manner consistent with policies intended to protect participant confidentiality and language in the Study protocol and informed consent form.

Human research participants

Policy information about studies involving human research participants and Sex and Gender in Research.

Reporting on sex and gender	Our findings are only generalizable to those who are female by sex and have menstruated at least once in life. Sex information was collected at enrollment by self-report. We did not implement any requirement on gender identity on participant's eligibility.
Population characteristics	A total of 794,282 menstrual cycles from 52,117 participants enrolled in the Apple Women's Health Study (AWHS) by December 31, 2021 were initially identified. After applying the exclusion criteria, a total of 12,608 participants were included in the final analysis. Mean age of eligible participants at baseline was 33 years old (SD = 8) and over 70% of the participants were White. Nearly 35% (N=4,379) of the participants were obese. Approximately 70% of the participants never smoked and 55% were nulliparous. As for alcohol use, 70% participants reported using alcohol use for no more than 4 times a month. A total of 59% participants had high education (i.e., college or graduate degree).
Recruitment	The Apple Women's Health Study is an ongoing, prospective digital cohort study. Users of the Apple Research app on their iPhone were eligible if they have ever menstruated at least once in life, live in the US, were at least 18 years old (at least 19 in Alabama and Nebraska, and 21 in Puerto Rico), and are able to communicate in English. Eligibility also required sole usage of their iCloud account or iPhone.
Ethics oversight	Written informed consent of participation is provided at enrollment. This study has been approved by the Institutional Review Board at Advarra (CIRB #PRO00037562) and has been registered in Clinicaltrials.gov (NCT04196595).

Note that full information on the approval of the study protocol must also be provided in the manuscript.

Field-specific reporting

Please select the one below that is the best fit for your research. If you are not sure, read the appropriate sections before making your selection.

☒ Life sciences ☐ Behavioural & social sciences ☐ Ecological, evolutionary & environmental sciences

For a reference copy of the document with all sections, see nature.com/documents/nr-reporting-summary-flat.pdf

Life sciences study design

All studies must disclose on these points even when the disclosure is negative.

Sample size	A total of 794,282 menstrual cycles from 52,117 participants enrolled in the Apple Women's Health Study (AWHS) by December 31, 2021 were initially identified. After applying the exclusion criteria, a total of 165,668 menstrual cycles from 12,608 participants were included in the final analysis.
Data exclusions	Data contributed by participants who did not meet the eligibility criteria, who reported menopause, who did not enroll and/or did not contribute a complete menstrual cycle by December 31, 2021, who reported history of PCOS, uterine fibroids, or hysterectomy were excluded.
Replication	We have not yet replicated our findings in other samples. However, we performed several sensitivity analyses to demostrate robustness of the results.
Randomization	This is an observational study. Therefore, no randomization was performed.
Blinding	This is an observational study. Therefore, no blinding was performed.

Reporting for specific materials, systems and methods

We require information from authors about some types of materials, experimental systems and methods used in many studies. Here, indicate whether each material, system or method listed is relevant to your study. If you are not sure if a list item applies to your research, read the appropriate section before selecting a response.

Materials & experimental systems

n/a	Involved in the study
☒	☐ Antibodies
☒	☐ Eukaryotic cell lines
☒	☐ Palaeontology and archaeology
☒	☐ Animals and other organisms
☒	☐ Clinical data
☒	☐ Dual use research of concern

Methods

n/a	Involved in the study
☒	☐ ChIP-seq
☒	☐ Flow cytometry
☒	☐ MRI-based neuroimaging
